# Recent advances in the investigation of the regulatory network underlying reactive nitrogen species-mediated tumorigenesis: molecular mechanisms and targeted therapeutic strategies

**DOI:** 10.1080/13510002.2025.2564593

**Published:** 2025-10-02

**Authors:** Yimao Wu, Zichang Chen, Xiaoyan Chen, Yinting Hu, Yunqi Ma

**Affiliations:** a School of Pharmacy, Binzhou Medical University, Yantai, People Republic of China; b Second Clinical Medical College, Guangdong Medical University, Dongguan, People Republic of China; c First Clinical Medical College, Guangdong Medical University, Zhanjiang, People Republic of China

**Keywords:** Reactive nitrogen species, tumorigenesis, tumor microenvironment, molecular mechanisms, targeted therapy, nitric oxide

## Abstract

Reactive nitrogen species (RNS) play a pivotal role in tumorigenesis through complex regulatory networks within the tumor microenvironment (TME). This review summarizes recent advances in understanding RNS-mediated mechanisms, focusing on core components and their concentration-dependent bidirectional effects on tumor cell proliferation, apoptosis, invasion, and metabolism. It explores RNS sources in the TME, including autonomous synthesis by tumor cells and secretion by immune cells (e.g., TAMs, TANs), and their modulation of key signaling pathways (e.g., PI3 K/Akt, NF-κB, HIF-1α). Additionally, the review discusses RNS-mediated regulation of immune responses and angiogenesis, highlighting their dual roles in promoting tumor progression and enabling immune evasion. Finally, it outlines potential clinical applications, such as RNS-targeted diagnostic probes and therapeutic strategies (e.g., iNOS inhibitors, NO donors), providing a foundation for precision oncology.

## 1. Introduction

In the decades of tumor biology research, reactive nitrogen species (RNS) – a family of redox-active molecules including nitric oxide (NO), nitrogen dioxide (NO₂), and peroxynitrite (ONOO^−^) – have evolved from being recognized as simple ‘toxic byproducts’ of inflammation to being acknowledged as pivotal endogenous regulators of tumorigenesis. Early studies in the 1990s first linked NO to tumor angiogenesis (e.g., via eNOS-mediated vascular dilation), yet subsequent work revealed conflicting roles: low NO concentrations drive tumor cell proliferation and immune evasion, while high NO/ONOO^−^ levels induce DNA damage and apoptosis – a duality that has long perplexed researchers [1]. This paradox arises from RNS’s intricate regulatory networks within the tumor microenvironment (TME), which involve crosstalk with immune cells (e.g., TAMs, TANs), stromal components, and key signaling pathways (e.g., PI3 K/Akt, NF-κB, HIF-1α) throughout tumor initiation, progression, and metastasis. Unraveling this complexity is therefore critical for advancing our understanding of tumor pathology and developing precise diagnostic and therapeutic strategies targeting RNS [2]. Recent research findings indicate that excessively active nitric oxide (NO), which is one of the RNS, signaling is closely linked to the advancement and prognosis of breast cancer [3]. Experimental interventions aimed at blocking inducible nitric oxide synthase (iNOS) have been shown to markedly impede the metastasis of breast cancer in mice [4]. Moreover, animal – based experiments have revealed that NO can suppress the proliferation of tumor cells [5]. Consequently, targeting reactive nitrogen and its regulatory mechanisms is regarded as a highly promising approach for cancer treatment [6,7]. Nevertheless, current strategies for targeting RNS face three critical unresolved challenges that hinder clinical translation. First, the bidirectional effects of RNS are not merely concentration-dependent but also context-specific – for example, NO derived from tumor cells promotes invasion via MMP activation, while NO from M1 macrophages exerts anti-tumor cytotoxicity – yet few studies have integrated these cell-type-specific roles into a unified framework [8]. Second, existing reviews on RNS and cancer often focus on isolated mechanisms (e.g., NO-mediated apoptosis or angiogenesis) and fail to systematically link RNS sources (tumor vs. immune cells), subcellular targeting (mitochondria vs. nuclei), and downstream metabolic/immune reprogramming in the TME [9]. Third, the crosstalk between RNS and other redox molecules (e.g., ROS, H₂S) in shaping TME homeostasis remains underdiscussed, limiting our ability to design combinatorial therapies [10]. To fill these gaps, this systematic review comprehensively synthesizes recent advances (2015–2025) in RNS biology within the TME, with a focus on: (1) the dual roles of core RNS components (NO, NO₂, ONOO^−^) in tumor cell functions and TME remodeling; (2) the integration of RNS sources, signaling pathways, and metabolic regulation; and (3) the translation of RNS-targeted strategies into diagnostic probes and therapies. By addressing these aspects, this review provides a novel, holistic framework to guide future RNS-focused tumor research and clinical applications. This article conducts a comprehensive and systematic review of the generation of RNS within the tumor microenvironment, their regulatory effects on tumor cells and the surrounding microenvironment, as well as their potential for clinical translation. It aims to offer a theoretical foundation for tumor research and practical applications.

## 2. Literature search methodology

To ensure comprehensive coverage of relevant studies, a systematic literature search was conducted across two major academic databases: PubMed and Web of Science. The search timeframe was restricted to articles published between January 2015 and April 2025 to focus on recent advancements. Key search terms included combinations of ‘reactive nitrogen species,’ ‘tumor microenvironment,’ ‘carcinogenesis,’ ‘regulatory mechanisms,’ ‘nitric oxide,’ ‘nitrosative stress,’ and ‘tumor progression’ . Additional keywords such as ‘RNS-mediated protein modification’ and ‘tumor microenvironment regulation’ were used to refine results.

Inclusion criteria prioritized peer-reviewed original research articles and comprehensive reviews published in English. Exclusion criteria included non-English publications, preprints, commentaries, letters to editors, and articles from journals listed in the academic warning list or not indexed in SCI. This screening process ensured the inclusion of high-quality, methodologically robust literature.

## 3. RNS overview and production within the tumor microenvironment

### 3.1. RNS core components and characteristics

RNS participate in the regulation of various processes within the tumor microenvironment through dual roles encompassing signal transduction and oxidative/nitrosative damage [11]. The functional diversity of RNS is largely determined by the specific core components involved. The primary RNS include NO, nitrogen dioxide (NO₂), peroxynitrite (ONOO^−^), among others [12]. NO is formed from L-Arg catalyzed by Nitric oxide synthase (NOS), and its half-life is only a few seconds [13]. NO is a highly reactive and diffusible free radical that functions as a physiological messenger, regulating numerous intracellular and extracellular signaling pathways. It is involved in critical physiological processes such as vasodilation, neurotransmission, cell migration, immune responses, and apoptosis, highlighting its potential significance within the tumor microenvironment [14]. NO₂, a prominent member of the RNS family, is predominantly generated through NO autoxidation (2 NO + O₂ → 2 NO₂) or enzymatic reactions catalyzed by myeloperoxidase (MPO) during inflammation [15,16]. Its strong oxidizing capacity enables it to rapidly nitrate tyrosine residues in proteins (forming 3-nitrotyrosine), induce lipid peroxidation, and cause DNA damage, thereby contributing to cytotoxicity, inflammatory responses, and tissue injury [17,18]. In the tumor microenvironment, elevated levels of NO₂ can synergize with ONOO^−^ to amplify oxidative and nitrosative stress, promoting apoptosis of cancer cells [19,20]. ONOO^−^, one of the most reactive RNS, is formed instantaneously through the reaction of NO with superoxide anion (O₂^−^•) in a 1:1 molar ratio. Despite its short half-life of approximately 10–20 milliseconds, it possesses sufficient permeability to penetrate lipid bilayers and can cleave iron-sulfur clusters, nitrate tyrosine residues, oxidize lipids, and damage DNA [21,22]. The core components of RNS in the tumor microenvironment including their formation, properties, and functions are summarized in Table 1.

**Table 1.** Core Components of RNS in the Tumor Microenvironment: Formation, Properties, and Functions.

Component	Formation Pathway	Half-life	Reactivity	Functions in the Tumor Microenvironment	References
NO	NOS-catalyzed oxidation of L-arginine (iNOS/eNOS/nNOS)	Seconds	Low (S-nitrosylation)	Signaling (pro-proliferation / angiogenesis); pro-tumorigenic at low concentrations	[14,23]
NO₂	NO auto-oxidation or MPO-catalyzed oxidation	Minutes	Moderate (tyrosine nitration)	Cooperates with ONOO^−^ to intensify oxidative stress and promotes apoptosis	[15,16]
ONOO^−^	1:1 reaction of NO with O₂^−^·	10–20 ms	High (oxidation / nitration)	DNA damage, mitochondrial dysfunction, pro-apoptosis / invasion	[21,22]

RNS molecules are characterized by unsaturated electronic structures that confer high chemical reactivity. They can modify biomacromolecules such as proteins (via nitration) and nucleic acids (via oxidation), thus participating in cell signaling and the regulation of cellular functions. Their main features include high reactivity, concentration-dependent bidirectional effects, targeted localization at subcellular sites, properties as signaling molecules, and a dependence on the local microenvironment.

#### 3.1.1. Concentration-dependent bidirectional effects

NO at extremely low concentrations can facilitate various physiological processes, including vasodilation, modulation of immune responses, neurotransmission, regulation of apoptosis, reproductive functions, gene transcription, mRNA translation, and post-translational modifications of proteins [24]. This suggests that at low levels, NO may contribute to tumor progression by exerting carcinogenic and anti-apoptotic effects. In the tumor microenvironment, reduced NO levels are often linked to substrate competition, such as increased consumption of L-arginine, or uncoupling of iNOS, resulting in vascular dysfunction [24,25]. Conversely, elevated NO concentrations exert inhibitory, nitrosative, and oxidative effects, leading to the formation of RNS that cause DNA damage, mitochondrial impairment, and higher rates of apoptosis [26]. At the tissue and organ levels, diminished NO bioavailability (low concentration) manifests as endothelial dysfunction, increased platelet aggregation, and enhanced inflammatory adhesion, thereby promoting pathogenic processes [27]. However, when NO levels are sufficiently restored either endogenously or pharmacologically, its effects shift toward protection, promoting vasodilation, anti-adhesive, and anti-thrombotic properties [27]. Additionally, at low concentrations, NO can subtly modify target proteins through S-nitrosylation or metal nitrosylation, activating signaling pathways such as soluble guanylate cyclase (sGC) that confer cellular protection [28,29]. As NO concentration increases, it rapidly reacts with superoxide anions to generate ONOO^−^, inducing intense oxidative and nitrosative stress, which can promote inflammation and apoptosis. This dualistic, dose-dependent nature of NO exemplifies its role as a double-edged sword in physiological and pathological contexts [30].

#### 3.1.2. Subcellular targeting and molecular mechanisms

RNS can localize to mitochondria, nuclei, and cell membranes, exerting their biological effects. Within mitochondria, complexes I and III of the electron transport chain are the primary sites of superoxide anion (O₂^−^·) generation during oxidative phosphorylation (OXPHOS). Elevated concentrations of NO rapidly react with superoxide anions produced at these sites, resulting in the formation of ONOO^−^ [14]. This potent oxidant can induce extensive oxidative damage to mitochondrial components, including the matrix, inner and outer membranes, and intermembrane space [14]. For instance, NO inhibits mitochondrial complexes I, II, and V by facilitating ONOO^−^ formation within mitochondria, which can irreversibly modify proteins through nitration of tyrosine residues [31]. Due to their small molecular weight, lipophilicity, and high reactivity, NO and its derivatives swiftly penetrate the lipid bilayer – within nanoseconds – targeting and modifying critical proteins. They preferentially nitrosylate cysteine residues located in active sites or transmembrane domains, thereby modulating the function of ion channels, receptors, and pumps in a reversible manner [32]. Moreover, NO can coordinate with metal centers such as heme iron or copper-zinc clusters through metal nitrosylation, transiently activating sGC or inhibiting cytochrome c oxidase, thereby enabling precise regulation of signaling pathways across membranes and intracellular compartments [33,34].

#### 3.1.3. High reactivity

RNS are characterized by their unsaturated electronic structures, such as NO and ONOO^−^, which confer high chemical reactivity. These species readily undergo nitration, oxidation, or nitrosylation reactions with cellular macromolecules, including proteins, nucleic acids, and lipids. NO specifically reacts with superoxide anion (O₂^−^•) to generate ONOO^−^, a potent oxidant that can be rapidly inactivated under certain conditions [35]. Peroxynitrite exerts significant oxidative and nitrosative stress effects within cells, inducing modifications such as S-nitrosylation of proteins, lipid peroxidation, and DNA nitration Nitric Oxide [36]. These chemical alterations can lead to profound effects on cellular integrity by disturbing protein conformation and function, damaging cell membrane structures, and causing DNA single-strand breaks. Such DNA damage activates poly(ADP-ribose) polymerase (PARP), which promotes DNA repair or, if the damage is extensive, initiates cell death pathways [25,37,38]. Notably, this RNS-triggered redox stress response follows a hormetic pattern: mild oxidative/nitrosative stress induced by low-to-moderate RNS levels does not exacerbate cellular damage but instead activates the Nrf2-associated resilience network. This network relies primarily on vitagenes – including heat shock proteins (Hsps), sirtuins, and Nrf2-regulated antioxidant genes – as core effectors to enhance cellular tolerance to subsequent stress, a mechanism with direct implications for antineurodegeneration. Calabrese et al. explicitly highlighted that hormetic activation of vitagenes via controlled redox signaling is pivotal for preserving neuronal integrity, as dysregulated redox homeostasis and impaired Nrf2-vitagenes signaling are key drivers of neurodegenerative disorders (e.g., Alzheimer’s and Parkinson’s diseases) [39]. This insight links RNS-mediated redox regulation (a central focus of this review) to broader neuroprotective mechanisms, extending the translational relevance of RNS research beyond oncology [40].

Moreover, peroxynitrite can target vital mitochondrial components across the matrix, inner and outer membranes, and intermembrane space, compromising mitochondrial function irreversibly. This impairment can inhibit mitochondrial respiration, further exacerbating cellular dysfunction and contributing to pathophysiological conditions associated with high reactivity of RNS [41].

#### 3.1.4. Signaling molecule properties

NO functions as a versatile signaling molecule that modulates cellular activities through both canonical and non-canonical pathways [25]. In the canonical pathway, NO exhibits relatively long-range signaling capabilities – diffusing approximately 50–100 μm to adjacent cells – and exerts its effects predominantly via the sGC-cyclic GMP (cGMP) pathway [42]. NO binds to the heme prosthetic group of sGC, activating the enzyme and promoting the conversion of GTP to cGMP [43,44]. The heme moiety in sGC is sensitive to NO-mediated nitrosylation, enabling activation even at low NO concentrations [44]. Elevated NO levels lead to increased cGMP production, which acts as a second messenger to activate cGMP-dependent protein kinase G (PKG) [45]. PKG then modulates a variety of cellular processes, including cardioprotection, inflammatory responses, phagocytic activity, inhibition of platelet aggregation, vasodilation, neural transmission, and calcium homeostasis. The degradation of cGMP by phosphodiesterases (PDE) maintains the signaling balance, with synthesis rates typically lower than degradation rates, thereby establishing a steady-state level dictated by sGC activity and PDE regulation [46].

Non-canonical NO signaling primarily involves covalent modifications of biomolecules by NO derivatives, such as S-nitrosylation, oxidation, nitration of proteins, and metal nitrosylation [47]. These post-translational modifications occur under physiological pH and serve to regulate protein structure and activity, impacting essential processes including gene transcription, DNA repair, cell growth, differentiation, and apoptosis [33]. Metal nitrosylation, in particular, involves NO binding to transition metals like heme within proteins, which can either activate or inhibit their functions – for example, activating sGC or inhibiting mitochondrial cytochrome C oxidase [33,48]. At low concentrations, NO generally exerts cytoprotective effects; however, at higher concentrations, it reacts with reactive oxygen species such as superoxide to form peroxynitrite. This reactive species can induce lipid peroxidation, protein nitrosylation, and other forms of oxidative and nitrosative stress, further influencing cellular signaling and inflammatory responses [47,48].

#### 3.1.5. Microenvironment dependence

NO within the tumor microenvironment is primarily produced by iNOS expressed in immune and tumor cells. The regulation of NO synthesis is influenced by hypoxia and inflammatory mediators, such as tumor necrosis factor-alpha (TNF-α) and interferon-gamma (IFN-γ). The availability and activity of NO are constrained by metabolic competition within the tumor microenvironment; notably, cancer cells compete for large amounts of L-arginine, which limits substrate availability for endothelial nitric oxide synthase (eNOS). This competition results in decreased NO production, contributing to abnormal blood vessel formation and function [49,50].

Prolyl hydroxylase domain (PHD) enzymes serve as critical negative regulators of hypoxia-inducible factor-1 alpha (HIF-1α) [51]. Under normoxic conditions, PHD hydroxylates HIF-1α, marking it for degradation. Conversely, hypoxia inhibits PHD activity, stabilizing HIF-1α, which then translocates to the nucleus where it collaborates with nuclear factor kappa-light-chain-enhancer of activated B cells (NF-κB) to promote iNOS transcription. This process leads to continuous NO synthesis, which reacts with mitochondrial reactive oxygen species (mtROS) to generate ONOO^−^. ONOO^−^ can further modify cellular components via S-nitrosylation, stabilizing HIF-1α and establishing a ‘hypoxia–HIF-1α–iNOS–RNS’ positive feedback loop [52].

Inflammatory cytokines such as TNF-α, interleukin-1 beta (IL-1β), and IFN-γ rapidly upregulate iNOS expression through activating the NF-κB, mitogen-activated protein kinase (MAPK), and Janus kinase-signal transducer and activator of transcription (JAK-STAT) pathways. TNF-α and IL-1β predominantly activate NF-κB signaling, whereas IFN-γ enhances iNOS transcription via the JAK-STAT pathway. These signaling cascades increase iNOS transcription efficiency by approximately 5 to 10-fold, leading to elevated production of RNS such as ONOO^−^ from NO and ROS interactions. These RNS further modify proteins through nitration, induce DNA damage, and activate inflammasomes, which in turn feed back into inflammatory signaling pathways. This forms an ‘inflammation–high RNS’ positive feedback loop, perpetually remodeling the tumor microenvironment [52,53].

### 3.2. Sources of RNS in the tumor microenvironment

The tumor metabolic microenvironment (TME) represents a highly intricate and dynamic ecosystem, composed of tumor cells, diverse stromal cell types, and immune cells, including tumor cells, cancer-associated fibroblasts (CAFs), stem cells, epithelial cells, mast cells, T lymphocytes, B lymphocytes, and macrophages [54,55]. These cellular populations exhibit differing capacities for nitric oxide synthase (NOS) expression, which are pivotal for the biosynthesis of NO. NO serves as the primary precursor for RNS within the TME [56,57].

NO is synthesized endogenously via three isoforms of nitric oxide synthase (NOS): neuronal nitric oxide synthase (nNOS or NOS1), inducible nitric oxide synthase (iNOS or NOS2), and endothelial nitric oxide synthase [58]. These enzymes operate in a tetrahydrobiopterin (BH4)-dependent manner, utilizing L-arginine and molecular oxygen as substrates. The enzymatic reaction requires oxygen and NADPH, converting L-arginine into L-citrulline and NO [59]. Each NOS isoform exhibits distinct expression patterns and activity durations, leading to different concentrations and roles of NO in biological systems [60,61]. For instance, eNOS and nNOS produce NO in a sustained manner over seconds to minutes at nanomolar concentrations, exerting roles in vasodilation and neurotransmission; meanwhile, NOS2 generates higher NO levels over extended periods, often associated with immune responses [60,62]. Specifically, nNOS predominantly resides in neural cells, iNOS is mainly expressed in immune cells such as macrophages and monocytes, and eNOS is primarily found in endothelial cells [63]. The expression patterns and roles of NOS isoforms in the tumor microenvironment are listed in Table 2.

**Table 2.** Expression Patterns and Roles of NOS Isoforms in the Tumor Microenvironment.

Isoform	Mainly Expressed In	NO Output Concentration	Duration	Role in Tumor Microenvironment	References
iNOS	Tumor cells, TAMs	Micromolar	Hours–days	High NO/ONOO^−^ levels; promotes apoptosis, invasion, and immune regulation	[60,61]
eNOS	Endothelial cells	Nanomolar	Seconds–minutes	Low NO concentration; promotes vascular dilation and angiogenesis	[60,62]
nNOS	Neuronal cells	Nanomolar	Seconds–minutes	Localized signaling; linked to neural infiltration in tumors (e.g., pancreatic cancer)	[63]

#### 3.2.1. Autonomous synthesis by tumor cells

Tumor cells can autonomously upregulate iNOS, which catalyzes the conversion of L-arginine into NO in the presence of molecular oxygen. The produced NO rapidly reacts with superoxide anion (O₂•^−^) to form ONOO^−^ and other RNS [64]. The expression of iNOS within tumors is characterized by persistent high levels, complex regulatory mechanisms, and tight metabolic coupling. Its activation is co-regulated by signals arising from hypoxia, inflammation, and metabolic cues within the microenvironment, making it a significant endogenous source of RNS in tumors [65,66].

Persistent high iNOS expression is maintained through epigenetic modifications such as H3K27 acetylation and promoter demethylation at the iNOS gene, ensuring continued transcription even after hypoxic or inflammatory stimuli subside [67]. Under normoxic conditions, PHDs hydroxylate HIF-1α, targeting it for recognition and degradation via the von Hippel–Lindau (VHL) tumor suppressor pathway [67]. Conversely, in hypoxic tumor regions, PHD activity is inhibited, leading to stabilization of HIF-1α, which translocates into the nucleus, heterodimerizes with Aryl Hydrocarbon Receptor Nuclear Translocator (ARNT), and binds to hypoxia-responsive elements (HREs) within the iNOS promoter (at approximately −1164/−1158 bp). This recruitment facilitates assembly of transcriptional co-activators like p300/CBP and enhances the binding of NF-κB, resulting in robust activation of iNOS transcription [67]. The sustained high levels of iNOS catalyze ongoing conversion of L-arginine into NO, which reacts with mtROS to produce ONOO^−^ and other RNS, further stabilizing HIF-1α via S-nitrosylation and establishing a positive feedback loop described as ‘hypoxia–HIF-1α–iNOS–RNS’. This loop amplifies RNS accumulation within the tumor microenvironment.

Under inflammatory stimuli, cytokines such as TNF-α and IL-1β bind to their respective receptors, TNFR1 and IL-1R1. This engagement activates downstream signaling cascades, including TRAF-mediated activation of IKKβ, which promotes nuclear translocation of NF-κB, and concurrently stimulates MAPK and PI3K-Akt pathways. NF-κB, along with transcription factors like IRF1 and STATs, binds to enhancer regions to markedly increase iNOS transcription, boosting NO synthesis [68,69].

**Figure 1.**
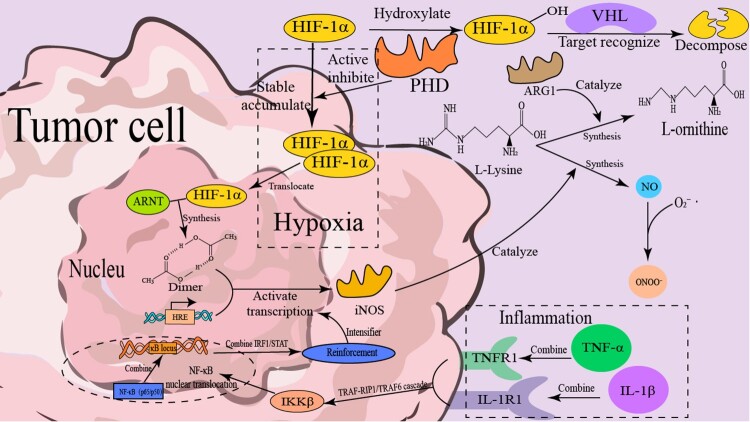
Scheme of HIF-1α- and inflammation-mediated RNS generation in tumor cells. Under hypoxia, stabilized HIF-1α binds ARNT, translocates to the nucleus, and activates iNOS transcription; iNOS then catalyzes NO production, which forms RNS. Under inflammation, factors like TNF-α/IL-1β activate iNOS via NF-κB, further promoting RNS production, enhancing inflammation, and regulating cellular metabolism.

Furthermore, tumor stem cells tend to co-express arginase-1 (ARG1) and iNOS, often depleting L-arginine by consuming it metabolically, which contributes to immunosuppression within the TME. Tumor cells upregulate ARG1 and iNOS to deplete extracellular L-arginine, causing T cell dysfunction and immune evasion [70,71]. L-arginine depletion by ARG1 can also induce NOS uncoupling, shifting NOS activity toward superoxide production (O₂•^−^), further exacerbating ONOO^−^ and other RNS levels and intensifying the immunosuppressive environment [71]. The mechanism by which tumor cells autonomously synthesize RNS is shown in Figure 1.

#### 3.2.2. Immune cell-mediated secretion

Innate immune cells, such as tumor-associated macrophages (TAMs) and neutrophils (TANs), are activated by tumor antigens, which trigger signaling through Toll-like receptors (TLRs). This activation involves the stimulation of NF-κB and MAPK pathways, exemplified by TLR4 recognition of lipopolysaccharide (LPS). Consequently, these pathways induce the expression of iNOS, leading to the catalytic conversion of L-arginine into substantial quantities of NO and other RNS, such as ONOO^−^ [72,73]. These RNS play roles in anti-tumor immune responses but can also be exploited by tumor cells to remodel the microenvironment. For instance, high levels of NO produced by M1 macrophages can exert cytotoxic effects by inhibiting mitochondrial respiration in tumor cells, while low NO concentrations may be harnessed by tumor cells to activate migration-related signaling pathways [74].

**Figure 2.**
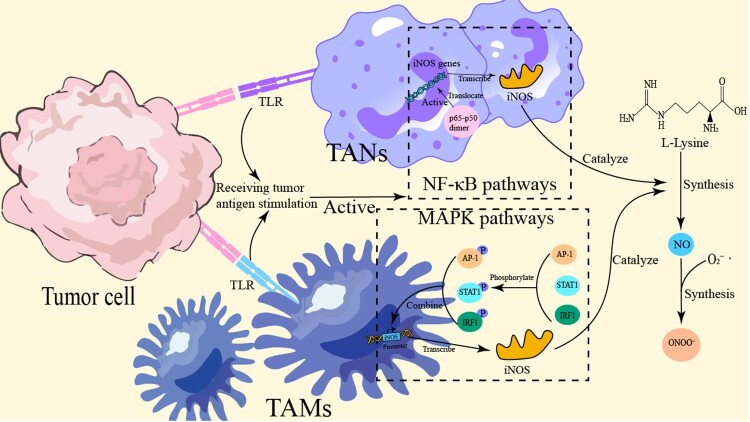
Scheme of immune cell RNS production. Antigen stimulation activates NF-κB (p65/p50) and MAPK pathways via Toll-like receptors (TLR), which further activate transcription factors AP-1 and IRF1. These factors bind the iNOS promoter to drive its transcription/translation; iNOS then catalyzes L-arginine conversion to NO, which reacts with oxygen to form RNS (e.g., peroxynitrite ONOO^−^). STAT1 phosphorylation also regulates iNOS synthesis, ultimately promoting RNS generation and enhancing immune cells’ bactericidal/inflammatory capacities.

The regulation of iNOS gene expression primarily involves MAPK and NF-κB signaling pathways. In the NF-κB pathway, the p65-p50 heterodimer translocates from the cytoplasm into the nucleus, where it promotes iNOS gene transcription [75]. In the MAPK pathway, transcription factors such as STAT1, IRF1, and AP-1 undergo phosphorylation, allowing them to translocate into the nucleus and bind cooperatively to the iNOS promoter, thereby enhancing transcription and subsequent NO production 46. Additionally, LPS can bind to TLR4 on macrophage membranes, further activating the MAPK and NF-κB pathways, which amplifies iNOS expression [76]. Studies by Hua Fan et al. on RAW264.7 macrophages demonstrated that LPS-induced activation of NF-κB p65 increases iNOS expression and promotes polarization toward the pro-inflammatory M1 macrophage phenotype [77]. Following LPS binding to TLR4, adaptor proteins MyD88 and TRIF initiate downstream cascades involving IRAK4, TRAF6, and TAK1, leading to phosphorylation and degradation of IκBα (thus releasing NF-κB p65/p50 into the nucleus) and the activation (phosphorylation) of MAPKs such as ERK1/2, p38, and JNK [78]. The mechanism by which immune cells mediate the secretion of RNS is shown in Figure 2. To concisely synthesize the core characteristics of RNS, their sources in the TME, and their potential clinical implications, Table 3 provides a summarized overview.

**Table 3.** Core Concepts and Clinical Relevance of RNS-Mediated Tumor Regulation Across Major Sections.

Section Number	Section Title	Core Concepts	Clinical Relevance
2	RNS Overview and Production within the Tumor Microenvironment	1. Core RNS components (NO, NO₂, ONOO^−^) exhibit distinct formation pathways, reactivities, and TME functions: NO acts as a signaling molecule (pro-angiogenic at low concentrations), NO₂ synergizes with ONOO^−^ to amplify oxidative stress, and ONOO^−^ induces severe biomacromolecule damage (e.g., DNA fragmentation, mitochondrial impairment). 2. RNS in the TME originate from two key sources: tumor cells autonomously upregulate iNOS via hypoxia (HIF-1α stabilization) and inflammation (NF-κB activation) feedback loops; immune cells (TAMs, TANs) secrete RNS via TLR-mediated iNOS induction.	Targeting iNOS (a primary RNS generator) or modulating RNS reactivity (e.g., scavenging ONOO^−^) could disrupt the TME redox balance, providing a foundational strategy for developing anti-tumor therapies.
3	RNS Regulates Tumor Cell Proliferation, Apoptosis, Invasion, Metastasis, and the Metabolic Microenvironment	1. Proliferation: NO exerts concentration-dependent effects – low NO (≤100 nM) activates PI3 K/Akt to promote G₁/S cell cycle transition, while high NO (≥500 nM) triggers p53-mediated G₁ arrest. 2. Apoptosis: ONOO^−^ drives mitochondrial dysfunction (cytochrome c release) and caspase cascade activation, whereas low NO inhibits apoptosis via S-nitrosylation of Bcl-2 or NF-κB-mediated upregulation of anti-apoptotic genes (e.g., Survivin). 3. Invasion/metastasis: RNS activates MMP-2/MMP-9 (via S-nitrosylation of catalytic cysteines) and Rho GTPases, while upregulating CXCR4 to guide tumor cell migration toward metastatic niches. 4. Metabolism: RNS reprograms TME metabolism by shifting to glycolysis (inhibiting mitochondrial complex IV) and enhancing glutamine catabolism (activating GLS1 via NF-κB/HIF-1α synergy).	Targeting RNS-mediated pathwayss (e.g., PI3 K/Akt, GLS1, MMPs) could simultaneously inhibit tumor growth, metastasis, and metabolic adaptation, supporting the design of multi-functional anti-tumor agents.
4	RNS Modulates Signaling Pathways That Influence the Tumor Microenvironment	1. Immune regulation: Low NO promotes immunosuppression (Treg differentiation, PD-L1 upregulation), while high NO/ONOO^−^ activates anti-tumor immunity (M1 macrophage polarization, DC maturation, enhanced NK cell cytotoxicity). 2. Angiogenesis: NO (via eNOS/VEGF axis) drives pro-angiogenic effects (endothelial cell proliferation, ECM remodeling); excessive ONOO^−^ disrupts vascular integrity (VE-cadherin degradation, pericyte loss) to facilitate tumor cell intravasation.	Combining NO donors (to boost anti-tumor immunity) with anti-angiogenic agents (e.g., VEGFR inhibitors) could counter RNS-mediated vascular dysfunction and synergize anti-tumor efficacy, informing combinatorial therapy protocols.
5	Clinical Translation Applications of RNS-Regulated Tumor Mechanisms	1. Diagnostic probes: Fluorescent probes (e.g., BODIPY for NO, rhodamine for ONOO^−^) enable high-sensitivity, tissue-specific RNS detection (e.g., mitochondrial NO in MCF-7 cells) with minimal interference from other ROS/RNS. 2. Therapeutic strategies: iNOS inhibitors (1400W, AG) reduce tumor growth/metastasis in TNBC models; NO prodrugs (e.g., JS-K) target GST-overexpressing tumors (e.g., CRPC) with low systemic toxicity; combining iNOS/BET inhibitors optimizes PDT by blocking NO-mediated ‘bystander cell invasion.’	RNS serve as both diagnostic biomarkers (e.g., iNOS expression, ONOO^−^ levels) and therapeutic targets, accelerating the translation of RNS-based strategies into precision oncology.

Collectively, the preceding section has established two core foundations of RNS biology in the TME: first, RNS (primarily NO, NO₂, and ONOO^−^) exhibit distinct formation pathways, reactivities, and concentration-dependent bidirectional effects – with low NO driving pro-tumor signaling (e.g., angiogenesis) and high NO/ONOO^−^ inducing anti-tumor damage (e.g., DNA fragmentation) [79]; second, RNS in the TME originate from two key cellular sources: tumor cells (via hypoxia-HIF-1α and inflammation-NF-κB feedback loops that upregulate iNOS) and immune cells (e.g., TAMs and TANs, which activate iNOS through TLR-mediated NF-κB/MAPK pathways) [80]. These RNS molecules, characterized by high reactivity and subcellular targeting (e.g., mitochondrial localization), do not act in isolation – instead, they directly modulate the biological behaviors of tumor cells that are critical for tumor progression [80]. The following section (Section 4) will build on this framework to systematically dissect how RNS regulate tumor cell proliferation (via PI3 K/Akt and AMPK pathways), apoptosis (through mitochondrial dysfunction and NF-κB modulation), invasion/metastasis (by activating MMPs and Rho GTPases), and metabolic reprogramming (via glycolysis and glutamine catabolism remodeling) – unraveling the functional consequences of RNS’s source- and concentration-dependent properties in shaping tumor cell fate.

## 4. RNS regulates tumor cell proliferation, apoptosis, invasion, metastasis, and the metabolic microenvironment

### 4.1. Intervention of tumor cell proliferation

NO influences tumor cell proliferation through a bidirectional regulatory mechanism: at low concentrations (≤100 nM), NO activates the PI3 K/Akt and AMPK signaling pathways, enhances cyclin D1 expression, accelerates the G₁/S phase transition, and thereby promotes tumor cell proliferation. Conversely, at high concentrations (≥500 nM), NO induces phosphorylation of p53 and inhibits cyclin-dependent kinase (CDK) complex activities, resulting in cell cycle arrest at the G₁ phase, and may even trigger apoptosis [81,82]. This concentration-dependent shift between pro-proliferative and anti-proliferative effects is modulated by tumor development stage and the degree of hypoxia within the microenvironment: in early-stage tumors, where oxygen partial pressure is approximately 1–5%, eNOS predominantly produces low levels of NO, supporting proliferation. In advanced hypoxic conditions (pO₂ < 1%), iNOS becomes significantly activated, often driven by HIF-1α and NF-κB. This results in a marked increase in NO, which can exert cytotoxic effects and improve chemosensitivity [36,83]. In studies of glioma, Bian and Murad observed that the NO/stromal cell-derived factor/gulcose-cGMP (NO/sGC/cGMP) signaling pathway plays a role in regulating cell proliferation and differentiation. They also noted that undifferentiated tumor cells and stem cells exhibit low levels of sGC. Consequently, elevated endogenous cGMP levels are negatively correlated with glioma cell proliferation and colony formation [84].

#### 4.1.1. PI3 K/Akt pathway

In tumor cells, the regulation of glycolysis involves several key pathways, notably the PI3 K/Akt pathway and other oncogenic signaling cascades such as Ras, HIF-1, and c-Myc pathways. The PI3 K/Akt pathway is frequently hyperactivated in human cancers [14], comprising phosphatidylinositol 3-kinase (PI3 K) and its downstream effector, protein kinase B (Akt/PKB). It functions as a central regulatory network governing cell growth, proliferation, survival, and metabolic processes. Among the oncogenic pathways that promote glycolysis in cancer cells, the PI3 K/Akt pathway is particularly prominent. Its activation occurs often in human malignancies, facilitating increased glycolytic flux.

The modulation of this pathway by NO depends on its concentration: at low NO levels, S-nitrosylation of the catalytic subunit of PI3 K at Cys895 relieves autoinhibition, thereby activating the pathway, which promotes cyclin D1 expression and facilitates the G₁/S cell cycle transition. Conversely, high NO concentrations, via ONOO^−^, oxidize or nitrosate critical cysteine residues in both PI3 K and Akt, rapidly inhibiting the pathway. This inhibition results in p53-mediated cell cycle arrest and apoptosis, establishing a redox switch – low NO levels promote tumor cell growth, while high NO levels inhibit proliferation through oxidative modifications modulated by hypoxia, iNOS, and RNS [85].

Furthermore, the activated PI3 K/Akt pathway enhances glucose uptake by promoting the translocation of glucose transporter 1 (GLUT1) to the plasma membrane, thereby increasing glycolytic flux to meet the energetic demands of proliferating tumor cells [64,86].

#### 4.1.2. AMPK pathway

RNS can nitrate subunits of mitochondrial ATP synthase, such as α, β, γ, and OSCP, leading to reduced ATP synthase activity [87]. This decline in ATP production can trigger a feedback activation of the AMP-activated protein kinase (AMPK) pathway and subsequent inhibition of the mammalian target of rapamycin (mTOR), resulting in cell cycle arrest [87,88]. At low NO concentrations, activation of the AMPK pathway occurs via the sGC-calcium/calmodulin-dependent protein kinase kinase 2 (CaMKK2) axis [89]. Tumor cells, often overexpressing CaMKK2, are particularly sensitive to this pathway. This mechanism, validated in endothelial cells exposed to NO derived from eNOS, involves NO binding to the heme group of sGC, which catalyzes the conversion of GTP to cGMP [89]. Elevated cGMP levels increase intracellular Ca²^+^ concentration, leading to extensive Ca²^+^ binding to calmodulin (CaM). The Ca²^+^-CaM complex activates CaMKK2, which phosphorylates and activates AMPKα at Thr172. Active AMPK then inhibits mTOR, causing cell cycle arrest [90,91].

**Figure 3.**
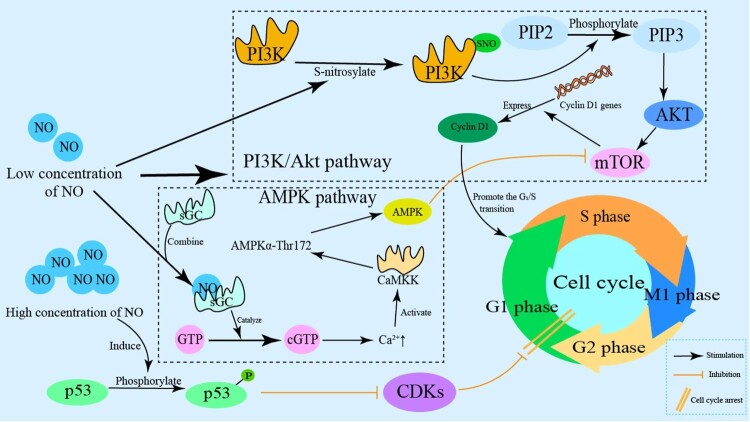
RNS-mediated regulation of tumor cell proliferation. RNS regulates tumor cell cycle/proliferation via multiple signaling pathways: Low NO activates the PI3 K/Akt pathway, promoting PIP2-to-PIP3 conversion and Akt activation; this further upregulates Cyclin D1 via mTOR, facilitating G1/S transition. High NO activates the AMPK pathway, where CaMKK-mediated AMPKα-Thr172 activation inhibits G1/S transition, causing cell cycle arrest. NO also inhibits proliferation by phosphorylating p53 and activating CDK inhibitors.

Within the tumor microenvironment, low NO levels produced by eNOS activate the sGC-CaMKK2-AMPK pathway, suppressing tumor cell proliferation. Conversely, high NO levels or ONOO^−^ oxidize critical cysteines in CaMKK2, interrupting this signaling cascade, thereby inactivating AMPK and resulting in the unchecked activation of mTOR. This redox-sensitive switch demonstrates that NO concentration determines whether the pathway exerts tumor-promoting or tumor-inhibiting effects, highlighting the dual role of NO in cancer biology through modulation of the sGC-CaMKK2-AMPK signaling axis [92]. The mechanism by which RNS intervenes in the proliferation of tumor cells is shown in Figure 3.

### 4.2. Regulation of tumor cell apoptosis

Depending on the cell type and the concentration of NO, NO can either induce or inhibit apoptosis across various cell populations. Tumor cells often develop tolerance to low concentrations of NO by upregulating anti-apoptotic proteins such as Bcl-2, whereas immune cells like T lymphocytes tend to be more susceptible to NO-induced apoptosis [93,94]. The NO produced by iNOS and neuronal nNOS exhibits cytotoxic properties largely due to its ability to react with superoxide anions to generate ONOO^−^, thereby advancing apoptotic processes [95]. The formation rate of peroxynitrite is influenced by the molar ratio of NO to superoxide (O₂^−^•) [94]. When NO and superoxide are present in near equimolar proportions, they rapidly react to produce peroxynitrite. As a highly reactive oxidant, peroxynitrite initiates a cascade of oxidative and nitrosative modifications within cells, damaging key biomolecules such as proteins, lipids, and DNA, which ultimately triggers apoptosis. This is evidenced in cell types including macrophages, pancreatic islets, thymocytes, and certain neurons [94]. DNA damage caused by peroxynitrite leads to an increase in p53 levels – a critical tumor suppressor – which in turn can upregulate p21, resulting in cell cycle arrest [96]. Moreover, peroxynitrite can damage mitochondrial DNA and activate the p53 pathway, thereby acting as a key initiator of tumor cell apoptosis [97].

**Figure 4.**
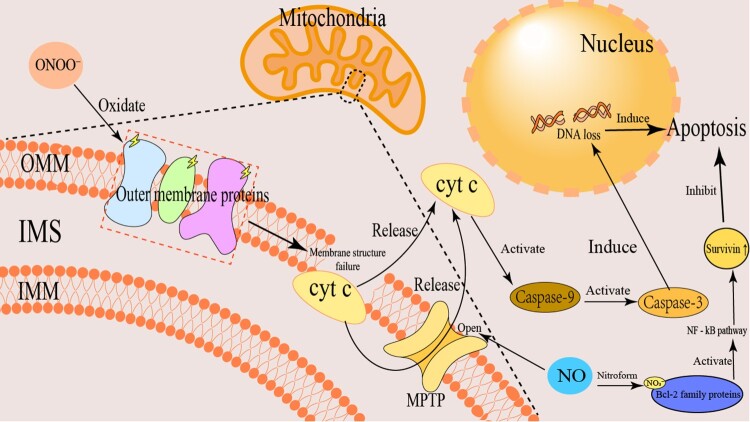
RNS regulation of tumor cell apoptosis. RNS (e.g., peroxynitrite ONOO^−^) oxidatively damage mitochondrial membranes, increasing permeability, releasing cytochrome c (cyt c), activating Caspase-3, and inducing apoptosis. Conversely, RNS may activate anti-apoptotic proteins (e.g., Survivin) via the NF-κB pathway to inhibit apoptosis. Additionally, RNS regulation of Bcl-2 family proteins affects mitochondrial membrane stability, further influencing apoptosis.

The regulation of tumor cell apoptosis by RNS exhibits a dual role. On the one hand, ONOO^−^ can promote apoptosis by oxidizing mitochondrial membrane proteins, facilitating cytochrome c release, and activating the caspase cascade[98]. On the other hand, NO can inhibit apoptosis via S-nitrosylation of Bcl-2 family proteins or by activating the NF-κB signaling pathway, which upregulates anti-apoptotic genes such as Survivin, thereby enabling tumor cells to evade apoptosis. Additionally, NO can induce modifications in the mitochondrial permeability transition pore (MPTP), where its opening results in the release of cytochrome c into the cytoplasm. This process triggers apoptotic signaling through the binding of cytochrome c to Apaf-1, leading to activation of caspase-9 and downstream executioner caspases 7 and 3, culminating in apoptosis [99]. The regulatory mechanism of RNS on tumor cell apoptosis is shown in Figure 4.

NO significantly influences cellular metabolism by modulating mitochondrial function. Cytochrome c oxidase, complex IV of the electron transport chain, accounts for approximately 90% of cellular oxygen consumption [100]. NO and ONOO^−^ contribute to mitochondrial regulation by oxidizing mitochondrial membrane proteins, promoting cytochrome c release, and inducing the opening of the mitochondrial permeability transition pore (PTP) via S-nitrosylation of critical subunits of ATP synthase (e.g., α-Cys251, β-Tyr345). This leads to loss of mitochondrial membrane potential and initiates mitochondrial-dependent apoptosis pathways, impairing the cell's capacity for oxidative phosphorylation [100,101]. The PTP comprises ATP synthase dimers, and its opening is governed by reactive oxygen and nitrogen species (ROS/RNS) [102]. Apoptosis mediated by mitochondria is primarily initiated by changes in mitochondrial outer membrane permeability (MOMP), resulting in the release of pro-apoptotic factors such as cytochrome c (Cyt c), Smac/DIABLO, apoptosis-inducing factor (AIF), and Endonuclease G, which activate caspases and promote cell death [103,104]. Notably, the likelihood of PTP opening is modulated by OXidants – higher RNS levels, especially when coupled with ROS, can lower the threshold for opening, thereby accelerating tumor cell apoptosis[105]. At low concentrations, NO can exert protective effects by S-nitrosylating and stabilizing proteins like Bcl-2, preventing their irreversible oxidation and thus inhibiting apoptosis [106].

In many cancers, the NF-κB transcription factor remains constitutively hyperactivated, leading to the upregulation of numerous downstream anti-apoptotic genes, including XIAP, Bcl-xl, Bcl-2, and YY1. These genes broadly influence tumor cell survival, proliferation, anti-apoptotic capacity, angiogenesis, and metastatic potential [107,108]. Therefore, inhibiting NF-κB activity can potentially reverse tumor progression and overcome drug resistance. Pharmacological agents, small molecule inhibitors, or siRNA targeting NF-κB can reduce tumor cell resistance to apoptosis, restoring sensitivity to chemotherapeutic and immunotherapeutic agents [109]. The impact of NO on NF-κB is concentration-dependent and bidirectional: high levels of NO inhibit NF-κB activity by obstructing the phosphorylation and degradation of its inhibitor I-κBα, thereby preventing NF-κB translocation into the nucleus and subsequent gene transcription [110]. Furthermore, elevated NO levels can scavenge reactive oxygen species like superoxide, impairing NF-κB activation [111]. By simultaneously inhibiting NF-κB nuclear translocation and neutralizing ROS, high-dose NO can synergistically suppress anti-apoptotic signaling pathways, repre-senting a promising strategy to reverse tumor drug resistance and enhance chemosen-sitivity and immunosensitization [107,112]. NO-mediated downregulation of NF-κB activity suppresses the expression of anti-apoptotic genes and limits pro-apoptotic gene prod-ucts [99]. Several NO donors have demonstrated efficacy in inhibiting NF-κB activity, thereby sensitizing resistant tumor cells to cytotoxicity

Immune cells can induce necrosis and apoptosis in target tumor cells. Cytotoxic lymphocytes such as cytotoxic T lymphocytes (CTLs) and natural killer (NK) cells recognize and bind specific receptors on the surface of tumor cells, become activated, and initiate apoptotic pathways. Post-activation, these effector cells utilize the perforin/granzyme system while secreting molecules like Fas ligand (Fas-L), tumor necrosis factor-related apoptosis-inducing ligand (TRAIL), and TNF-α. These molecules bind to their respective receptors on target cells through extrinsic pathways, mediating either necrosis or apoptosis [113]. Tumor cells possess varying degrees of resistance to such cytotoxic stimuli, often reflected in their anti-apoptotic threshold. Several strategies, including the use of non-specific and specific sensitizers, aim to lower this threshold, rendering tumor cells more vulnerable to therapeutic-induced apoptosis [110]. RNS can enhance immune-mediated cytotoxicity by activating NK cell and CTL responses, promoting dendritic cell maturation and antigen presentation, and amplifying anti-tumor immunity. Studies have shown that NO donors can be employed to diminish tumor cell resistance, facilitating apoptosis [110]. At micromolar concentrations, NO exhibits direct tumoricidal effects; NO produced by macrophages, NK cells, endothelial cells, and Kupffer cells inhibits tumor growth through regulation of enzymes like aconitase and ribonucleotide reductase, exerting cytostatic and cytotoxic effects [114].

NO also influences tumor cell apoptosis by reversing Fas-mediated apoptosis tolerance. Evidence suggests that IFN-γ can restore FasL-driven apoptotic sensitivity in tumor cells by inducing iNOS expression and subsequent NO production [115]. Mechanistically, NO donors such as S-nitroso-N-acetylpenicillamine (SNAP) and DETA-NONOate can S-nitrosylate the transcriptional repressor YY1, impairing its ability to bind the Fas promoter silencer region, thereby enhancing Fas expression and sensitizing tumor cells to FasL-induced apoptosis [110]. This process involves the synergistic activation of both the extrinsic (type I) and intrinsic (type II) apoptosis pathways, providing a basis for RNS-based immunosensitization therapies.

NO also modulates tumor cell resistance to TNF-α-induced apoptosis. Constitutive activation of NF-κB is a central mechanism underlying resistance to TNF-α. Unlike TNF receptor 1 (TNF-R1), TNF receptor 2 (TNF-R2) preferentially activates NADPH oxidase via TRAF2, leading to reactive oxygen species (ROS) generation and subsequent NF-κB activation, which inhibits apoptosis [116]. Interestingly, IFN-γ can reverse this resistance by stimulating iNOS expression, resulting in NO production. NO reacts with ROS to generate ONOO^−^, which impairs ROS-mediated NF-κB activation, thereby restoring tumor cell sensitivity to TNF-α-induced apoptosis [117]. This effect can be blocked by NOS inhibitors such as L-NMMA, while NO donors like SNAP can mimic IFN-γ's sensitizing action.

Furthermore, NO can overcome TRAIL resistance by upregulating death receptor 5 (DR-5). NO donors such as DETA-NONOate inhibit the repressive binding of YY1 to the DR-5 promoter, leading to increased DR-5 expression and restoring sensitivity to TRAIL-induced apoptosis [118]. This involves activation of both mitochondrial (type I) and death receptor (type II) apoptotic pathways, indicated by caspase-9 and caspase-3 activation, as well as PARP cleavage – an effect synergistic with chemotherapy combined with NF-κB inhibition [112].

### 4.3. Promote tumor invasion and metastasis

#### 4.3.1. Degradation of the basement membrane

RNS modulate the cysteine residues of matrix metalloproteinases (MMPs), transforming these enzymes into potent mediators of tumor invasion. Specifically, ONOO^−^ or low concentrations of NO reversibly S-nitrosylate the catalytic cysteine residues of matrix metalloproteinase-2 (MMP-2) and matrix metalloproteinase-9 (MMP-9) (such as Cys102 in MMP-2 and Cys99 in MMP-9). This modification removes the propeptide's inhibitory effect on the zinc ions in the enzyme's active site, facilitating the activation of the zymogen during secretion. The propeptide domains of MMPs coordinate with the catalytic Zn²^+^ ion through histidine residues; S-nitrosylation of Cys102/Cys99 disrupts this coordination, resulting in premature enzyme activation [119]. Additionally, the same modification occurs at cysteine residues within the heme-binding region, stabilizing the enzyme’s conformation and markedly enhancing its capacity to cleave the triple-helical regions of type IV collagen (Gly-X-Y repeats), thereby significantly increasing basement membrane degradation [120].

RNS can further upregulate the transcription of MMP genes, promote increased secretion of MMP proteins, and sustain invasive capabilities. NO enhances MMP-2 and MMP-9 expression while concurrently downregulating tissue inhibitors of metalloproteinases (TIMPs)−2 and −3, thereby facilitating tumor invasion [25,121]. ONOO^−^ nitrates tyrosine residues, such as Tyr42 of IκBα, increasing its affinity for SCFβ-TrCP ubiquitin ligase, promoting rapid ubiquitination and degradation of IκBα. This process releases NF-κB p65/p50 dimers, allowing their translocation into the nucleus where they bind to the κB sites at positions −600/−602 bp of the MMP-9 promoter. The activated NF-κB complex co-activates histone acetyltransferases CBP/p300 and PCAF, which acetylate histones and further promote MMP-9 transcription, thereby enhancing basement membrane degradation and tumor invasion [122].

#### 4.3.2. Activation of migration-related signaling pathways

Rho family GTPases, including RhoA, Rac1, and Cdc42, are small molecular switches that regulate cytoskeletal dynamics and cell migration, which are essential for tumor cell motility. RNS, particularly NO and ONOO^−^, target the conserved phosphorylation loop (GXXXXGK[S/T]C) of these GTPases [123]. They oxidize nearby cysteine residues (Cys16/Cys20) via two-electron oxidation or radical-mediated nitrosylation, significantly increasing the GDP dissociation rate by approximately 500–600 times – effectively mimicking the action of guanine nucleotide exchange factors (GEFs). This results in the immediate activation of Rho GTPases. Similarly, ONOO^−^ oxidizes Cys340 of p115-RhoGEF, enhancing its GEF activity by two to threefold, which further amplifies RhoA-GTP levels. RNS-mediated phosphorylation by protein kinase C (PKC) can also activate p115-RhoGEF, establishing a positive feedback loop that sustains GTPase activation [124]. These activated Rho GTPases promote the formation of stress fibers through Rho-associated kinase (ROCK), activate PAK-Arp2/3 complexes to form actin meshworks, and stimulate WASP-mediated actin filament assembly – all orchestrating cytoskeletal reorganization essential for cell migration [125].

In addition, RNS upregulate the expression of CXCR4 via an ‘oxidation-phosphorylation-transcription’ pathway [126]. CXCR4 is highly expressed in various tumors and directs cell migration, invasion, and metastasis along the SDF-1α gradient. Low-dose NO, through sGC-cGMP-CaMKK2 signaling, promotes phosphorylation of AMPK, relieving negative feedback on mTOR and maintaining PI3 K/Akt pathway activity. Conversely, high-dose ONOO^−^ nitrates IκBα, releasing NF-κB, which then forms an enhancer complex with HIF-1α. This complex binds to HRE/κB sites in the CXCR4 promoter, recruiting co-activators p300/CBP, leading to a significant increase in CXCR4 transcription. Elevated CXCR4 expression guides tumor cells toward metastatic niches along SDF-1α gradients [126–128]. Furthermore, PTEN acts to suppress the PI3 K/AKT pathway [129], however, NO inhibits NF-κB, Snail, and YY1, resulting in PTEN downregulation and subsequent suppression of PTEN-mediated PI3 K/AKT signaling, thereby influencing proliferation and apoptosis resistance pathways in tumor cells [129,130].

### 4.4. Reprogramming the tumor metabolic microenvironment

The TME is pivotal in tumor progression and treatment outcomes, significantly influencing tumor cell proliferation, invasion, and metastasis [131]. Within the two central metabolic pathways – glucose and glutamine metabolism – RNS, particularly NO and ONOO^−^, dynamically modulate mitochondrial respiration, glycolysis, the tricarboxylic acid (TCA) cycle, and lactate efflux in a concentration-dependent manner. These processes supply essential energy for tumor cell growth and metastatic spread [132]. Additionally, the accumulation of lactate contributes to the formation of an acidic and immunosuppressive niche, further supporting tumor survival and immune evasion. RNS also stimulate CAFs to upregulate glucose transporters, thereby enhancing their lactate production and delivery to tumor cells, ultimately establishing a metabolic symbiosis that fuels tumor metabolism. Furthermore, RNS induce post-translational modifications – such as oxidation and nitrosation – on critical metabolic enzymes including ATP synthase, hexokinase 2 (HK2), and glutaminase 1 (GLS1). These modifications help construct a reversible and dynamic metabolic-immune regulatory axis, presenting a novel and promising target for therapeutic intervention [133].

#### 4.4.1. Regulating carbohydrate metabolism

The accumulation of lactate within the tumor microenvironment has been shown to impair the cytokine-producing capacity of tumor-infiltrating T cells and NK cells. This impairment hampers their activation and diminishes their ability to mediate tumor immune surveillance. Additionally, the highly acidic conditions resulting from excess lactate promote immune evasion by recruiting and activating immunosuppressive cell populations, including M2-like macrophages, regulatory T cells (Tregs), and myeloid-derived suppressor cells (MDSCs) [132]. NO can inhibit mitochondrial respiratory chain complex IV (cytochrome c oxidase), leading to a 30–50% reduction in mitochondrial ATP production. This inhibition shifts cellular energy metabolism towards glycolysis, thereby enhancing aerobic glycolysis in tumor cells and increasing lactate secretion in the tumor microenvironment, which further facilitates metabolic reprogramming of tumor cells [14,134]. Hypoxia is another key driver of metabolic reprogramming within the tumor microenvironment. NO can inhibit proline hydroxylase-mediated degradation of HIF-1α. Even in the presence of oxygen, NO can induce hypoxic responses by stabilizing HIF-1α, thus promoting hypoxia-driven metabolic adaptations in tumor cells [135].

Moreover, RNS-dependent regulation of ATP synthase activity also plays a role in tumor metabolic reprogramming. High expression of ATP synthase inhibitory factor 1 (IF1) is characteristic of the Warburg phenotype in tumors, and NO-mediated nitrosylation of IF1 significantly influences its activity, allowing transient restoration of oxidative phosphorylation. Studies demonstrate that tumor cells overexpress IF1 to inhibit ATP synthase, thereby forcing reliance on glycolysis for energy (the Warburg effect) [136,137]. NO can S-nitrosate IF1, specifically at Cys65, which reduces IF1’s binding affinity for the ATP synthase alpha subunit. This modification decreases inhibition, promotes pro-teasomal degradation of IF1, and briefly restores oxidative phosphorylation – an adap-tive response to hypoxic conditions[138].This dynamic regulation through S-nitrosylation constitutes a potential target for metabolic interventions aimed at the RNS axis in tumor cells. The modification at Cys65 decreases IF1’s interaction with ATP synthase, promoting its degradation, and adjusting tumor metabolism accordingly.

#### 4.4.2. Regulating glutamine metabolism

Glutaminase 1 (GLS1) is localized on the outer surface of the mitochondrial inner membrane and functions as the rate-limiting enzyme in glutamine catabolism. It catalyzes the hydrolysis of glutamine into glutamate and ammonia [139]. GLS1 cooperates with the mitochondrial inner membrane transporter SLC1A5 to facilitate the uptake of cytoplasmic glutamine into mitochondria, where it is rapidly metabolized. Subsequently, glutamate is converted into α-ketoglutarate (α-KG) by glutamate dehydrogenase (GDH) or various aminotransferases, feeding into the TCA cycle to support the energetic, reducing equivalent, and biosynthetic demands of rapidly proliferating tumor cells [139]. RNS can activate GLS1, enhancing the glutamine breakdown into α-KG, thus providing carbon skeletons, nitrogen, and the reducing power necessary for tumor cell biosynthesis [140,141]. For instance, ONOO^−^ nitrates IκBα, resulting in the release and nuclear translocation of NF-κB p65, which in turn binds to the GLS1 promoter. Simultaneously, NO inhibits PHD, stabilizing HIF-1α. Both NF-κB and HIF-1α synergistically bind to specific promoter regions: the κB site, located at -300 bp, and the hypoxia-responsive element (HRE), located at -150 bp; these sites are separated by 150 bp, allowing the formation of an enhancer loop that markedly enhances transcriptional activity and upregulates GLS1 expression by approximately fold [140].

## 5. RNS modulates signaling pathways that influence the tumor microenvironment, impacting tumor progression and immune responses

### 5.1. RNS regulates immune-related signaling pathways

#### 5.1.1. Immunosuppression

NO plays a critical role in regulating the apoptosis and survival of a broad spectrum of immune cells, including dendritic cells, mast cells, NK cells, macrophages, monocytes, Kupffer cells, microglia, eosinophils, and neutrophils. Its effects are highly dependent on concentration and cellular context. At low concentrations, NO promotes cell survival by activating anti-apoptotic proteins and cGMP signaling pathways. Conversely, at high concentrations, NO induces apoptosis through mitochondrial pathways, activation of caspases, or modulation of signaling loops such as NF-κB, Snail, and YY1 [142,143]. NO can inhibit phosphorylation of Cys466 downstream of TCR kinase ZAP70 via S-nitrosylation, leading to a reduction in IFN-γ transcription. Additionally, NO promotes Treg differentiation, upregulates immune checkpoint molecules (e.g., programmed cell death ligand 1 (PD-L1)), and fosters an immunosuppressive tumor microenvironment [144]. Bogdan et al. demonstrated that NO influences mast cell survival by regulating mitochondrial function. Initial studies have shown that macrophages produce NO derivatives such as NO₂^−^ and NO₃^−^ when stimulated with recombinant IFN-γ and LPS, and that NO inhibits iron-dependent enzymes (such as uricase and mitochondrial complex I/II) by binding to iron-containing cofactors. This results in decreased activity of uricase and mitochondrial complexes I and II within cytotoxic macrophages. These inhibitory effects are dependent on the presence of NO [145]. Prashant Trikha et al. co-cultured MDSCs from cancer patients with autologous NK cells to evaluate antibody-dependent cellular cytotoxicity (ADCC) against trastuzumab-coated tumor cells. Their findings revealed that MDSCs significantly impair NK cell-mediated ADCC in a dose-dependent manner [146]. Importantly, the use of the iNOS inhibitor L-NIL restored NK cell ADCC activity (p < 0.01), indicating that NO suppresses NK cell function and that combined therapy with iNOS inhibitors may overcome trastuzumab resistance mediated by MDSCs, offering a promising approach for treating HER2-positive breast cancer.

#### 5.1.2. Immune activation

Lck and Syk are key kinases located downstream of the NK cell NKG2D receptor. Their enhanced S-nitrosylation facilitates binding to the immunoreceptor tyrosine-based activation motif (ITAM), thereby activating the PLCγ2-Ca²^+^ signaling pathway [147]. RNS can enhance NK cell cytotoxicity. At low concentrations, NO and ONOO^−^ reversibly S-nitrosylate kinases such as Lck and Syk, which strengthens their interaction with the ITAM motifs downstream of NKG2D and natural cytotoxicity receptors (NCRs), and promotes phosphorylation of PLCγ2. This leads to accelerated polarized release of granzyme B and perforin, thereby improving the killing efficiency of NK cells. In contrast, high concentrations of these species inhibit this process through nitrosative stress [147,148]. In a 4-hour 51Cr release assay, treatment with 0.5 µM NO donor DETA-NONOate increased the specific lysis rate of human peripheral blood NK cells against K562 cells from 35% to 62%, and nearly doubled CD107a degranulation. This enhancing effect was completely abolished by the iNOS inhibitor L-NMMA, confirming that exogenous low-dose NO can directly amplify NK cell cytotoxicity [149]. Finisguerra et al. co-cultured NK cells with ONOO^−^ generated by mouse N1-type neutrophils stimulated with lipopolysaccharide (LPS) and interferon-beta (IFN-β), and observed a fourfold increase in NK cell IFN-γ secretion and a 60% reduction in B16F10 lung metastatic nodules. These antitumor effects were significantly attenuated following treatment with the ONOO^−^ scavenger FeTPPS [150]. This experiment demonstrates that RNS derived from N1-type neutrophils can activate the killing function of NK cells both in vitro and in vivo.

**Figure 5.**
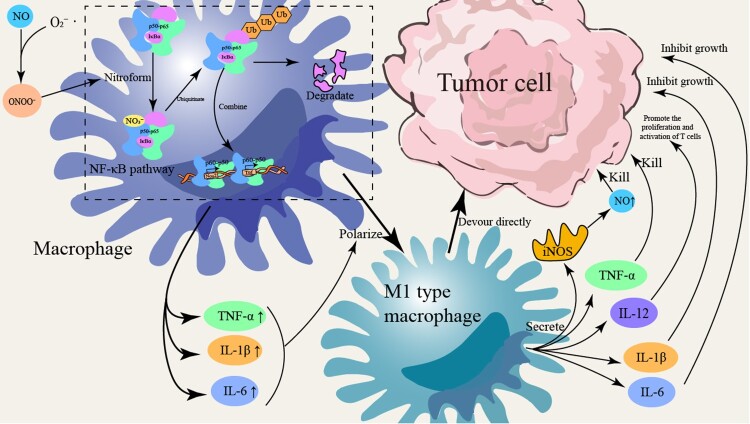
RNS-mediated immune regulation and anti-tumor effects in the TME. NO and ONOO^−^ modify NF-κB pathway proteins (e.g., IκBα) via ubiquitination to inhibit tumor cell growth/proliferation. Meanwhile, RNS activate the NF-κB (p50-p65) pathway to promote T cell activation/proliferation. Additionally, RNS induce M1 macrophage polarization, prompting secretion of pro-inflammatory cytokines (TNF-α, IL-1β, IL-6, IL-12) to enhance immune responses and directly phagocytose tumor cells, supporting tumor suppression and immune regulation.

RNS can also promote the maturation of dendritic cells (DCs). Within the tumor microenvironment, the NO-mediated nuclear factor kappa-light-chain-enhancer of activated B cells (NF-κB) pathway is involved in M1 macrophage polarization. Recent studies indicate that oscillatory versus sustained NF-κB signaling elicits distinct transcriptional responses in mouse macrophages [151]. Upon activation by TLR ligands, DCs highly express iNOS, which generates NO that reacts with superoxide anions to form ONOO^−^, thereby initiating the NF-κB pathway [151]. NO rapidly combines with superoxide anions (O₂^−^) to form the highly reactive ONOO^−^, which can nitrate IκBα, leading to its degradation and the subsequent release of NF-κB (p65/p50) into the nucleus. The p65 subunit then synergistically binds with interferon regulatory factor 5 (IRF5) and signal transducer and activator of transcription 1 (STAT1) at the promoters of Nos2 and Tnf, thereby upregulating the expression of pro-inflammatory cytokines such as TNF-α, interleukin-1 beta (IL-1β), and interleukin-6 (IL-6). This promotes macrophage polarization toward the M1 phenotype [152,153]. M1-type macrophages exert antitumor effects in the early stages of tumorigenesis by secreting pro-inflammatory factors and phagocytosing tumor cells, thus inhibiting tumor growth [154]. M1 macrophages can also induce tumor cell apoptosis by producing large amounts of NO/RNS through modification of the death receptor pathway, mitochondrial cytochrome c release, and inhibition of NF-κB [152]. In contrast, M2-type macrophages and their subsets produce lower levels of NO/RNS. Hypoxic conditions and microenvironmental factors such as NO/RNS stabilize transcription factors such as HIF and its family members, activate the mitogen-activated protein kinase ERK1/2 pathway, and promote the expression of vascular endothelial growth factor (VEGF) and other pro-angiogenic molecules [154]. The immune regulation and anti-tumor mechanisms of RNS in the tumor microenvironment are shown in Figure 5.

RNS can enhance antigen presentation. Low doses of NO or ONOO^−^ can S-nitrosylate the NF-κB inhibitory protein IκBα, accelerating its degradation and promoting NF-κB nuclear translocation. This upregulates the expression of major histocompatibility complex class II (MHC-II), CD80, CD86, and interleukin-12 (IL-12) on the surface of DCs, thereby providing T cells with both signal 1 (MHC-peptide antigen presentation) and signal 2 (costimulatory signals) [155]. Furthermore, RNS can promote the cross-presentation of antigens to CD8^+^ T cells. The mild oxidative stress induced by RNS increases cross-presentation efficiency by threefold, accounting for 40% of total antigen presentation. This facilitates the escape of exogenous antigens into the cytoplasm and their subsequent loading onto MHC-I molecules via the proteasome–endoplasmic reticulum pathway, thereby achieving cross-presentation [156]. At the same time, ONOO^−^ moderately disrupts the lysosomal pH gradient, reducing excessive antigen degradation and increasing the abundance of MHC-I-peptide complexes [156]. Additional studies have shown that CD4^+^ T cell help can induce downregulation of glutaredoxin in plasmacytoid dendritic cells (pDCs), leading to oxidative imbalance and lipid peroxidation. This promotes the efficient activation of OT-I CD8^+^ T cells by soluble ovalbumin (OVA) via the phagosome–cytoplasm pathway, an effect that can be reversed by antioxidant treatment [157].

Enhancement of NO-mediated immunotherapy is a promising strategy, and certain biologics have been shown to elevate NO levels. For instance, antibodies targeting the overexpressed PD-L1 in cancer cells – such as atezolizumab, avelumab, and durvalumab – or antibodies blocking the PD-1 receptor on immune cells including natural killer T cells, dendritic cells, B cells, CD4^+^ and CD8^+^ T cells – can benefit from NO modulation as part of combination therapy[158]. These immunotherapeutic approaches may indirectly enhance patient NO release. Moreover, since the transcription factor YY1 further upregulates PD-L1 expression and NO is known to inhibit YY1, NO donors may further improve the efficacy of PD-1/PD-L1 immunotherapy [159].

### 5.2. RNS regulates angiogenesis-related signaling pathways

#### 5.2.1. Pro-angiogenesis

NO can serve as the final effector molecule in angiogenesis signaling pathways. For instance, pro-angiogenic factors such as VEGF activate protein kinase B (Akt) via VEGF receptor 2 (VEGFR2). With the assistance of phosphoinositide-dependent kinase 1 (PDK1), Akt phosphorylates Ser1177 on eNOS, thereby enhancing NO production [160]. This represents the upstream signaling cascade through which NO exerts its pro-angiogenic effects. The downstream effects of NO-mediated pro-angiogenesis encompass four key processes: promotion of endothelial cell proliferation and migration, upregulation of pro-angiogenic factor expression, extracellular matrix (ECM) remodeling, and mobilization and homing of endothelial progenitor cells (EPCs) [161–163]. NO can activate sGC, leading to increased synthesis of cGMP, which in turn activates PKG and initiates the MAPK pathway [164]. This signaling axis underlies NO-mediated endothelial cell proliferation and migration – both of which are essential for angiogenesis. Seung Namkoong et al. demonstrated that eNOS gene knockout in mice resulted in the absence of vascular sprouting in aortic rings compared to wild-type controls, providing direct evidence that the eNOS/NO pathway is indispensable for prostaglandin E2 (PGE2)-mediated angiogenesis [163].

NO also inhibits PHD-containing protein-mediated hydroxylation of HIF-1α, thereby stabilizing HIF-1α under normoxic conditions and upregulating VEGF expression, which further enhances angiogenesis [165]. This mechanism illustrates how NO promotes the expression of pro-angiogenic factors. Notably, VEGF can reciprocally stimulate NO synthesis through the aforementioned upstream pathway, establishing a positive feedback loop. Additionally, NO activates matrix metalloproteinase-13 (MMP-13) and membrane-type 1 matrix metalloproteinase (MT1-MMP), facilitating ECM degradation, which is critical for endothelial cell migration and lumen formation [166]. MMP-13 preferentially degrades type II collagen, while MT1-MMP activates MMP-2, collectively contributing to basement membrane degradation in a synergistic manner [167]. These processes collectively achieve ECM remodeling, a pivotal step in pro-angiogenesis. Furthermore, NO promotes the mobilization of EPCs to ischemic regions, where they participate in angiogenesis and tissue repair [168].

**Figure 6.**
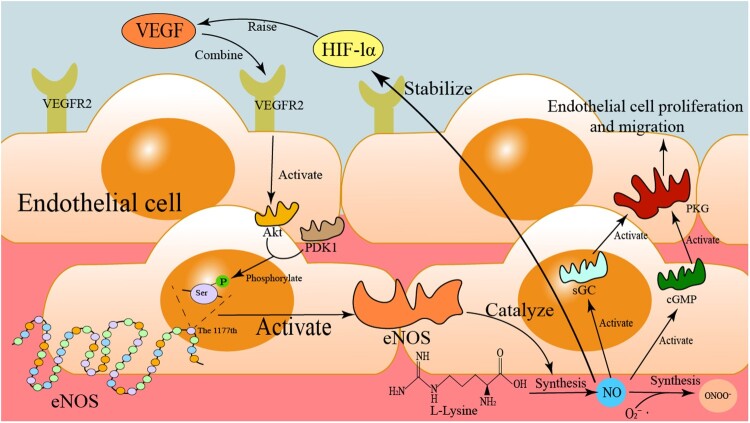
RNS-driven angiogenesis mechanism. Under hypoxia, stabilized/activated HIF-1α binds VEGFR2 to promote endothelial cell proliferation/migration. NO activates sGC to produce cGMP, which activates PKG; PKG then promotes eNOS phosphorylation/activation via the Akt-PDK1 pathway, ultimately enhancing NO synthesis. NO further reacts with oxygen to form ONOO^−^, supporting angiogenesis and cellular metabolism.

The regulatory role of NO in microcirculation is also closely linked to the metabolic adaptations of tumor cells under conditions that threaten their survival [169]. For example, eNOS-derived NO plays a crucial role in maintaining microvascular function. Elevated uptake of L-arginine by cancer cells reduces the availability of this substrate for eNOS, leading to diminished NO production and the subsequent formation of microvessels with abnormal architecture, structure, and function [49]. NO is recognized as an endothelium-derived relaxing factor, continuously synthesized from the amino acid L-arginine and oxygen by eNOS in endothelial cells, and it exerts significant effects on all vascular wall cell types [27]. As a potent vasodilator, NO also provides multiple vascular protective benefits, including inhibition of platelet aggregation, prevention of leukocyte or monocyte adhesion to the endothelial surface, suppression of vascular smooth muscle cell proliferation and migration, and protection against vasculitis [165,170]. For instance, NO promotes arteriolar dilation, thereby increasing blood flow to tumors and inducing angiogenesis necessary for tumor progression [171]. The mechanism of RNS-induced angiogenesis is shown in Figure 6.

#### 5.2.2. Induction of vascular dysfunction

Excessive generation of RNS, particularly ONOO^−^, can directly impair the structural integrity and functional stability of vascular endothelial cadherin (VE-cadherin) through mechanisms involving oxidative stress and tyrosine phosphorylation. This leads to the internalization and subsequent degradation of VE-cadherin, as well as its dissociation from β-catenin. Such disruptions compromise the integrity of interendothelial adherens junctions, resulting in increased vascular permeability, the formation of intercellular gaps, and ultimately facilitating the transendothelial migration (also known as intravasation) of tumor cells into the circulatory system [172]. For instance, VEGF induces VE-cadherin tyrosine phosphorylation and internalization via VEGFR2-mediated activation of Src kinase. This process disrupts the vascular endothelial barrier, thereby promoting tumor cell extravasation and metastasis [172]. Furthermore, the reduction or functional disruption of VE-cadherin is closely associated with tumor-induced increases in vascular hyperpermeability, which enhances the opportunity for tumor cells to enter the bloodstream [172].

Excessive RNS, especially ONOO^−^, can also comprehensively dismantle the structural and functional integrity of the tumor vasculature through multiple synergistic mechanisms. On one hand, RNS directly nitrate VE-cadherin and induce its tyrosine phosphorylation, leading to internalization and degradation. These alterations disrupt interendothelial adherens junctions, promote the formation of intercellular gaps, and significantly elevate vascular permeability [173]. On the other hand, RNS inhibit the PDGF-BB/PDGFR-β signaling pathway, which triggers apoptosis and detachment of pericytes. This results in the loss of the protective pericyte ‘sheath’ and the supporting basement membrane, leading to the development of tortuous and dilated vascular lumens along with basement membrane disruption [174–176]. The resulting structurally disorganized and highly permeable tumor vasculature not only exacerbates the local hypoxic and acidic microenvironment but also captures circulating tumor cells (CTCs) by upregulating the expression of adhesion molecules such as E-selectin, ICAM-1, and the chemokine CXCL12. Additionally, the extraluminal deposition of extracellular matrix components such as fibronectin and Tenascin-C contributes to the formation of an immunosuppressive premetastatic niche. Collectively, RNS-driven vascular abnormalities accelerate tumor metastasis across the entire metastatic cascade – from the intravasation of tumor cells into the circulation, through hematogenous dissemination, to their eventual extravasation and colonization at distant sites [177]. These processes underscore the pivotal role of RNS in the tumor invasion-metastasis cascade. Moreover, similar mechanisms, including VE-cadherin internalization and its dissociation from the β-catenin complex, have also been implicated in enhancing vascular permeability, thereby further facilitating the dissemination of tumor cells within the circulation and their establishment at distant metastatic sites [178].

## 6. Clinical translation applications of RNS-regulated tumor mechanisms

### 6.1. Development of novel diagnostic and prognostic biomarkers

#### 6.1.1. NO probes

Current methodologies for detecting NO encompass a range of techniques, including electron paramagnetic resonance (EPR) spectroscopy, chemiluminescence, electrochemical assays, and fluorescent probe methodologies. Among these approaches, the fluorescent probe technique has emerged as the most efficacious for detecting NO within biological systems, owing to its superior sensitivity, exceptional selectivity, and user-friendly operational characteristics [179]. Liu et al. engineered a BODIPY-based fluorescent probe specifically designed for NO detection [180]. In its unbound state, the probe demonstrates minimal emission solely at 570 nm, attributable to the photoinduced electron transfer (PET) process. However, upon NO introduction, the emission peak at 570 nm is markedly intensified due to the inhibition of the PET mechanism. This probe exhibits a high degree of specificity for NO, demonstrating a linear correlation between fluorescence enhancement and NO concentration within the range of 1.7–8.3 mM, and boasts a detection limit of 5.0 × 10^−^⁷ M. The utility of this probe has been validated through its successful application in detecting exogenous NO in both RAW264.7 macrophages and human retinal pigment epithelial cells [180]. Similarly, Nagano et al. developed a near-infrared luminescent probe tailored for NO detection [181]. This particular probe incorporates Yb³^+^ ions but remains non-emissive under standard conditions due to the PET process occurring within the ligand structure. In the presence of NO, o-diaminophenyl is transformed into triazole, thereby obstructing the PET process and facilitating energy transfer from 2,7-dichlorocalcein to Yb³^+^. This results in robust luminescence emission at 980 nm, accompanied by a substantial Stokes shift of 480 nm. The probe displays high selectivity for NO, with a detection limit of 90 nM [181]. Furthermore, Jin and Xiao et al. devised a rhodamine-based fluorescent probe for NO detection. In its native state, the probe neither absorbs light at 555 nm nor emits fluorescence. However, upon the addition of NO within the concentration range of 1–10 mM, the probe exhibits an absorption band at 555 nm and an emission peak at 585 nm, both of which demonstrate a linear relationship with NO concentration. The probe has a detection limit of 4.0 nM. Notably, other substances such as ONOO^−^, H₂O₂, ¹O₂, NO₃^−^, NO₂^−^, OH^−^, divalent cations, glutathione, cysteine, dehydroascorbic acid, and ascorbic acid do not induce any fluorescence alterations, underscoring the probe's high specificity for NO [182]. This rhodamine-based probe is equipped with a mitochondrial-targeting triphenylphosphine moiety and has been effectively employed for the real-time tracking of mitochondrial NO in MCF-7 cells. The mitochondrial targeting efficiency of the probe is enhanced by fivefold, achieving a signal-to-noise ratio of 30:1, thereby enabling the real-time monitoring of burst-like mitochondrial NO generation in MCF-7 cells under hypoxic conditions.

#### 6.1.2 Peroxynitrite probe

Peroxynitrite (ONOO^−^) is a highly reactive oxidant formed through the reaction between nitric oxide (NO) and the superoxide anion (O₂^−^). As a key member of the RNS, it plays a critical role in various pathological and physiological processes. The development of fluorescent probes capable of specifically detecting ONOO^−^ is of significant importance for the diagnosis and mechanistic study of related diseases. These fluorescent probes typically interact with ONOO^−^ via specific chemical reactions, leading to detectable changes in fluorescence signals [182]. For instance, some probes operate based on mechanisms such as intramolecular charge transfer (ICT) or excited-state intramolecular proton transfer (ESIPT). Upon reaction with ONOO^−^, these processes are often disrupted, resulting in observable fluorescence changes [183]. Tang et al. developed a near-infrared organic selenium-based fluorescent probe for the detection of ONOO^−^ [184]. This probe exhibits a maximum emission wavelength at 800 nm when excited at 770 nm. Upon the addition of ONOO^−^, a reaction product (110-I) is formed, leading to a decrease in fluorescence intensity at 800 nm. The sensing mechanism involves the oxidation of divalent selenium by ONOO^−^, resulting in the formation of SeQO bonds [184]. This probe demonstrates high selectivity for ONOO^−^, with a detection limit in the range of 0–5.0 mM, and has been successfully employed for multi-cycle real-time imaging of oxidative stress and reductive repair processes in RAW264.7 macrophage cells. Yoon and Ryu designed a ratiometric fluorescent probe for detecting ONOO^−^ [184]. By incorporating an OCl^−^-specific scavenging group (such as a thioether), interference from hypochlorite (OCl^−^) was minimized to below 5%. When excited at 475 nm, the probe emits fluorescence at 635 nm. Upon addition of ONOO^−^, a new fluorescence peak emerges at 515 nm, while the intensity at 635 nm decreases. The fluorescence intensity ratio (FI515 nm/FI635 nm) exhibits a linear correlation with ONOO^−^ concentration in the range of 0–25 mM, and the detection limit is as low as 49.7 nM. This probe shows high selectivity for ONOO^−^, with minimal interference from other reactive oxygen species (including NO, O₂^−^, H₂O₂, OCl^−^, OH^−^, and t-BuOO^−^) and biological thiols (such as Cys, Hcy, and GSH). However, at very high concentrations (e.g., 4200 equivalents), OCl^−^ can induce similar ratio changes [184]. This ratiometric probe has been successfully used for the real-time imaging of endogenous ONOO^−^ in live cells. In RAW264.7 cells, the generation of ONOO^−^ results in a reduction of red fluorescence and the appearance of green fluorescence. Yang et al. developed a turn-on type fluorescent probe for detecting ONOO^−^ [185]. Upon addition of ONOO^−^, the probe exhibits a significant enhancement in green fluorescence at 535 nm under excitation at 517 nm. The detection limit of this probe for ONOO^−^ is as low as 10 nM. When exposed to other biologically relevant reactive oxygen and nitrogen species (such as H₂O₂, O₂^−^, NO, and ROO^−^), the fluorescence intensity of probe 120 remains largely unchanged. Notably, even HOCl and OH^−^ only induce negligible increases in fluorescence, highlighting the probe’s high selectivity [185]. The fluorescence enhancement mechanism is attributed to ONOO^−^-induced oxidative cleavage, which releases a luminescent rhodamine-based fluorophore. This probe demonstrates low cytotoxicity and has been effectively applied for imaging ONOO^−^ in multiple cell lines.

#### 6.1.3. Nitroxyl probes

Nitroxyl (HNO), the one-electron reduced and protonated derivative of NO, exhibits distinct biological effects compared to NO. Research indicates that under specific conditions, NOS can directly generate HNO, and interconversion between NO and HNO can occur via superoxide dismutase. Due to its high reactivity, nitroxyl spontaneously dimerizes to form nitrous oxide (N₂O) upon dehydration, prompting the development of probes capable of sensitive and selective detection of this RNS [182]. For instance, Nakagawa et al. engineered a fluorescent probe for HNO detection [186]. Upon exposure to HNO, the probe displayed a marked enhancement in fluorescence emission at 526 nm when excited at 491 nm. Other RNS and reactive oxygen species – including ONOO^−^, NO₂^−^, NO₃^−^, H₂O₂, and OCl^−^ – had negligible impact on the probe’s fluorescence, demonstrating its high selectivity for HNO [186]. The proposed reaction mechanism involves HNO-induced deprotection of 2-(diphenylphosphino)benzoate, leading to the release of the fluorescent compound 123-I. Sun et al. developed a FRET-based ratiometric fluorescent probe for HNO detection [187]. This probe emitted fluorescence at 470 nm; however, in the presence of HNO, the fluorescence intensity at 517 nm increased while that at 470 nm decreased (excitation wavelength: 415 nm). This shift was attributed to FRET from the coumarin to the fluorescein fluorophore, which was released through oxidative ring opening. Within the concentration range of 0–100 mM, the fluorescence intensity ratio (FI517 nm/FI470 nm) exhibited a linear correlation with HNO concentration, and the detection limit was determined to be 1.4 mM. Other reactive oxygen species and RNS – such as H₂O₂, OH^−^, t-BuO^−^, O₂^−^, OCl^−^, benzoyl peroxide, and NO₂ – did not interfere with the probe’s ability to detect HNO, underscoring its high selectivity for HNO [187]. A summary of fluorescent probes for detecting RNS in biological systems is provided in Table 4.

**Table 4.** Summary of Fluorescent Probes for Detecting RNS in Biological Systems.

Probe Type	Target	Ex / Em (nm)	LOD	Selectivity (Anti-interference)	Application (Cell Line / Tissue)	References
BODIPY probe	NO	Ex∼500, Em ∼570	5.0 × 10^−^⁷ M	Resistant to ROS/RNS	RAW264.7 macrophages, retinal cells	[180]
NIR probe	NO	Ex∼770, Em ∼980	90 nM	High (NO-specific)	In-vivo imaging (deep-tissue penetration)	[181]
Rhodamine probe	ONOO^−^	Ex∼517, Em ∼535	10nM	Resistant to HOCl/·OH	Various tumor cell lines (mitochondria-targeted)	[185]
FRET probe	HNO	Ex∼415, Em∼470/517	1.4mM	Resistant to ROS	Real-time HNO dynamics in living cells	[187]

### 6.2. Targeted therapeutic targets and intervention signaling pathways

#### 6.2.1. Inhibition of RNS generation

In various cancers – including colorectal cancer, gastric cancer, hepatocellular carcinoma, melanoma, breast cancer, leukemia, prostate cancer, esophageal cancer, and cervical cancer – elevated expression of iNOS is significantly associated with poor patient survival outcomes [188,189]. Consequently, iNOS expression can serve as a prognostic biomarker indicative of unfavorable survival [190]. iNOS inhibitors have the potential to block excessive NO synthesis in tumor cells and have shown promise in preclinical studies by inhibiting tumor proliferation and enhancing sensitivity to radiotherapy and chemotherapy [191]. The iNOS-selective inhibitor 1400W exhibits over 100-fold greater selectivity for iNOS compared to eNOS and effectively suppresses NO generation in tumor cells. Treatment of cells with high iNOS expression – such as triple-negative breast cancer (TNBC) – with 1400W (a highly selective iNOS inhibitor), L-NAME (a relatively selective eNOS inhibitor), or L-NMMA (a pan-NOS inhibitor) resulted in reduced cell proliferation, migration, and mammosphere formation [191]. In a TNBC xenograft model, administration of L-NAME and L-NMMA to mice significantly attenuated tumor growth [191]. Another iNOS-specific inhibitor, AG, was used in athymic nude mice bearing TNBC xenografts and was found to reduce both tumor growth and metastatic burden [191]. AG achieved a 50% reduction in tumor size in the TNBC model, slightly lower than the 65% reduction observed with 1400W, but with reduced toxicity [191].

Emerging therapeutic strategies include the development of prodrugs conjugated to antigens, which leverage tumor enzyme activity – such as glutathione S-transferase (GST) – and binding to tumor-specific antigens. Antigen aptamer-labeled NO donors can enhance prostate cancer-specific delivery while minimizing systemic toxicity [192]. JS-K is an example of such an NO prodrug, which is activated by GST overexpressed in prostate cancer cells. JS-K has been shown to inhibit prostate cancer growth and promote apoptotic pathways through the ubiquitin-proteasome pathway. As an effective prodrug, JS-K generates NO exclusively in the presence of tumor enzymes, making it a promising candidate for prostate cancer treatment, particularly in castration-resistant prostate cancer (CRPC). The NO prodrug JS-K has also been reported to reduce intracellular androgen receptor levels via the Wnt signaling pathway, supporting the notion that high-dose NO delivery can disrupt nuclear receptor function within cells [193].

#### 6.2.2. Intervention in downstream signaling pathways

For signaling pathways regulated by RNS, such as the MAPK and NF-κB pathways, the development of small molecule inhibitors – such as MEK inhibitors – can effectively block abnormal proliferation and metastatic signaling in tumor cells. These inhibitors work synergistically with RNS modulation to enhance therapeutic efficacy.

YY1 is a transcriptional repressor that inhibits the expression of PD-L1 by binding to its promoter region. However, S-nitrosylation of YY1 can alleviate this inhibitory effect, thereby enhancing PD-L1 expression and potentially compromising the effectiveness of immune checkpoint blockade therapy. Targeting the S-nitrosylation of YY1 offers a molecular intervention strategy to modulate NO-mediated effects and reverse tumor immune evasion mechanisms [194,195]. Preclinical studies have demonstrated that NO inhibits YY1-DNA binding activity and upregulates the expression of Fas, a cell surface death receptor. This upregulation en-hances the sensitivity of tumor cells to Fas ligand (FasL)-mediated apoptosis induced by CTLs and NK cells[196,197].These findings provide a novel rationale for combining NO donors with immunotherapy to improve anti-tumor immune responses.

#### 6.2.3. Latest strategies for optimizing photodynamic therapy (PDT) regimens by combining iNOS/BET inhibitors

In addition to traditional approaches targeting RNS generation pathways, recent research has introduced a novel combinatorial strategy involving the use of NOS activity inhibitors, such as GW274150 and bromodomain and extra-terminal motif (BET) epigenetic inhibitors (such as JQ1 applied to PDT) [198]. This dual inhibition approach addresses both PDT-induced NO upregulation and its downstream ‘bystander effect.’ In this phenomenon, NO diffuses from irradiated to non-irradiated tumor areas, activating MMP-9 in non-targeted cells and thereby enhancing their migratory capabilities. The combined inhibition of iNOS and BET pathways effectively blocks this NO-mediated enhancement of migration and invasion in bystander cells [199].

Specifically, this strategy simultaneously inhibits iNOS/NO-mediated anti-apoptotic signals and pro-invasive signaling in directly targeted tumor cells, while also impeding the proliferation and migration of adjacent non-irradiated cells that are influenced by NO diffusion. Furthermore, the combined inhibition of iNOS and BET pathways reduces the nitrosylation of ATP synthase, particularly at the Cys251 residue within its α subunit. Attenuating nitrosylation at this site leads to a decreased frequency of PTP opening in mitochondria, resulting in a significant reduction – approximately 40% – in mitochondrial ROS release [200].

This integrated intervention (iNOS/BET dual inhibition) not only markedly diminishes the risks of tumor recurrence and metastasis following PDT but also establishes a new translational framework for solid tumor combination therapy [201]. Beyond this PDT-optimized strategy, three novel RNS-targeted therapeutic approaches have recently emerged with promising preclinical potential, addressing key limitations of existing methods (e.g., systemic toxicity, poor tissue specificity). First, ‘RNS-responsive nanocarriers’ – engineered to release payloads (e.g., iNOS inhibitors, NO donors) upon exposure to TME-specific RNS (e.g., ONOO^−^) or redox cues (e.g., high H₂O₂) – have shown enhanced tumor accumulation: for example, ONOO^−^-cleavable polymeric nanoparticles loaded with 1400W (iNOS inhibitor) achieved a 4.2-fold higher tumor-to-normal tissue drug ratio in TNBC xenografts compared to free 1400W, reducing off-target endothelial toxicity [202]. Second, ‘ONOO^−^ scavengers combined with immune checkpoint inhibition’ (e.g., anti-PD-1) address RNS-driven immunosuppression: preclinical studies in melanoma models demonstrated that FeTPPS (a selective ONOO^−^ scavenger) reduced Treg infiltration by 35% and enhanced CD8^+^ T cell cytotoxicity, synergizing with anti-PD-1 to shrink tumors by 60% – a effect attributed to diminished ONOO^−^-mediated PD-L1 upregulation on tumor cells [203]. Third, ‘dual-targeting drugs co-modulating RNS and metabolic pathways’ (e.g., iNOS/GLS1 inhibitors) leverage RNS’s role in glutamine reprogramming: a novel hybrid molecule (iNOS inhibitor-GLS1 inhibitor conjugate) suppressed both NO production and glutamine catabolism in CRPC cells, reducing cell proliferation by 72% compared to single-target inhibitors – capitalizing on the synergistic crosstalk between NF-κB (activated by RNS) and GLS1 transcription [204]. These strategies expand the translational toolkit for RNS-targeted therapy, bridging TME-specificity, immune modulation, and metabolic coordination to overcome current clinical barriers.

**Figure 7.**
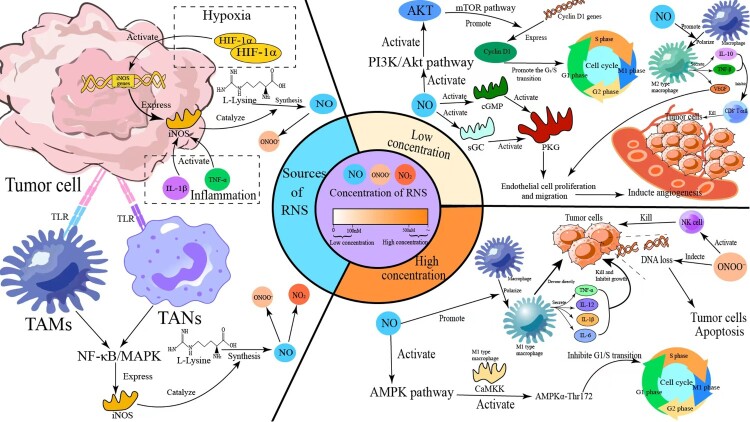
Integrated scheme of RNS sources, concentration-dependent effects, and downstream tumor regulation in the TME. Left panel: Major RNS (NO, ONOO^−^) sources include tumor cells and immune cells. Tumor cells generate RNS via hypoxia-stabilized HIF-1α (activates iNOS for L-arginine-to-NO conversion), amplified by inflammatory cytokines (IL-1β, TNF-α). TAMs/TANs produce RNS via TLR-mediated NF-κB/MAPK activation (upregulates iNOS). Middle panel: Concentration dictates function: Low RNS (e.g., NO ≤100 nM) activates PI3 K/Akt/mTOR (upregulates Cyclin D1, promotes G₁/S transition), induces M2 polarization (IL-10-mediated immunosuppression), and activates endothelial sGC/cGMP/PKG (drives proliferation/migration/angiogenesis). High RNS (e.g., NO ≥500 nM or excessive ONOO^−^) triggers AMPK (CaMKK-mediated AMPKα-Thr172 phosphorylation, blocks G₁/S), induces apoptosis (DNA damage/mitochondrial dysfunction), polarizes M1 macrophages (secretes TNF-α/IL-12/IL-6/IL-1β), and enhances NK cell cytotoxicity. Right panel: Low RNS promotes tumor progression (cell cycle acceleration, immunosuppression, angiogenesis); high RNS inhibits tumors (apoptosis, pro-inflammatory immunity, cell cycle arrest) – highlighting RNS concentration as a key regulator of tumor biology.

To explicitly bridge RNS-mediated molecular mechanisms with ongoing translational efforts, Table 5 summarizes key RNS-targeted therapeutic agents, their mechanisms of action, current development stages (preclinical or clinical), and available trial identifiers, highlighting their clinical relevance. Figure 7 integrates RNS sources, concentration-dependent bidirectional effects, and their downstream molecular and cellular mechanisms, providing a comprehensive conceptual framework for understanding RNS-mediated tumor progression and suppression.

**Table 5.** Translational Progress of RNS-Targeted Therapeutic Agents for Cancer Treatment.

Therapeutic Agent Type	Agent Name	Target/Mechanism of Action	Development Stage	Cancer Type Targeted	Clinical Trial Identifier
iNOS Inhibitors	1400W	Selective inhibition of iNOS; reduces NO/ONOO^−^ production to suppress tumor proliferation and metastasis	Preclinical (in vivo)	Triple-negative breast cancer (TNBC), colorectal cancer	N/A (preclinical)
iNOS Inhibitors	AG	Selective iNOS inhibition; decreases tumor growth and metastatic burden with lower systemic toxicity	Preclinical (in vivo)	TNBC	N/A (preclinical)
NO Prodrugs	JS-K	GST-activated NO release; inhibits androgen receptor signaling and induces apoptosis via ubiquitin-proteasome pathway	Phase I Clinical Trial	Castration-resistant prostate cancer (CRPC)	NCT00373877
NO Donors	SNAP	Exogenous NO donor; S-nitrosylates YY1 to upregulate Fas, sensitizing tumor cells to immune-mediated apoptosis	Preclinical (in vitro/in vivo)	Melanoma, non-small cell lung cancer (NSCLC)	N/A (preclinical)
iNOS/BET Dual Inhibitors	GW274150 + JQ1	GW274150 (iNOS inhibitor) + JQ1 (BET inhibitor); blocks NO-mediated ‘bystander invasion’ and reduces mtROS release post-PDT	Preclinical (in vitro)	Solid tumors (e.g., glioma, breast cancer)	N/A (preclinical)

## 7. Future perspectives on RNS-targeted cancer therapy

### 7.1. Core challenges and unmet needs in RNS-targeted therapy

Despite significant advancements in unraveling RNS-mediated regulatory networks within the tumor microenvironment (TME) and their initial translational success, such as the development of iNOS inhibitors and NO prodrugs – critical challenges persist that hinder the clinical application of RNS-targeted strategies [205]. The most formidable barrier is the inherent bidirectional complexity of RNS: at low concentrations, NO drives pro-tumor processes including angiogenesis, Treg differentiation, and immune evasion, while high levels of ONOO^−^ induce anti-tumor effects such as DNA damage and NK cell activation, yet current interventions (e.g., fixed-dose iNOS inhibitors) fail to adapt to dynamic TME changes like fluctuating hypoxia, inflammatory cytokine surges, or L-arginine competition between tumor and immune cells, resulting in inconsistent therapeutic outcomes [206]. Another key unmet need is the lack of single-cell resolution in RNS metabolism analysis; bulk measurements of RNS levels mask significant heterogeneity among tumor subclones, immune cell subsets (e.g., M1 vs. M2 TAMs), and stromal cells, making it impossible to identify cell-type-specific RNS regulatory circuits that could be targeted for precision therapy [207]. Additionally, the cross-talk between RNS and other redox-active molecules (e.g., ROS, H₂S) in the TME remains poorly characterized, limiting the understanding of how RNS integrate into broader redox networks to influence tumor progression and immune responses.

### 7.2. Priority research directions to address RNS-mediated complexity

To overcome the challenges outlined above, future research should focus on three interconnected areas that build on existing insights into RNS biology. First, developing TME-responsive precision targeting systems is essential to tailor RNS modulation to real-time TME conditions – for example, engineering hypoxia-activated iNOS inhibitors that are only activated in hypoxic tumor regions (where iNOS is overexpressed and RNS levels are excessive) or pH-sensitive NO donors that release NO specifically in the acidic TME niches occupied by aggressive tumor cells, which would minimize off-target toxicity to normal tissues while maximizing anti-tumor efficacy [208]. Second, integrating advanced single-cell spatial imaging tools with multi-omics approaches will enable a more detailed understanding of RNS dynamics: combining multiplexed NO fluorescent probes (e.g., rhodamine-based ONOO^−^ probes) with single-cell RNA sequencing and spatial metabolomics can map RNS distribution, metabolic pathways, and signaling networks across individual components of the TME, identifying ‘RNS oncogenic hotspots’ such as tumor subpopulations dependent on low NO for proliferation or immune cells that secrete RNS to drive immunosuppression [209]. Third, designing synergistic combinatorial regimens to counter RNS bidirectionality will be critical – for instance, pairing iNOS inhibitors with anti-PD-L1 antibodies to reduce immunosuppressive low-RNS signaling while amplifying anti-tumor immunity, combining NO prodrugs with GLS1 inhibitors to disrupt RNS-mediated glutamine metabolism in tumor cells [210], or co-administering ONOO^−^ scavengers with anti-angiogenic agents (e.g., VEGFR2 inhibitors) to mitigate RNS-driven vascular dysfunction and prevent tumor metastasis [211].

### 7.3. Strategies to accelerate translational progress of RNS-targeted therapies

Translating preclinical RNS research into clinical practice requires addressing practical barriers that have slowed the development of other redox-targeted therapies. Optimizing TME-penetrating drug delivery systems is a key first step: developing lipid nanoparticles conjugated to TME-specific ligands (e.g., CXCR4 aptamers or CD44 antibodies) can enhance the accumulation of RNS-targeted agents in tumors, as these ligands bind to receptors overexpressed on tumor cells and stromal cells, improving tissue penetration while reducing systemic toxicity – a strategy that has already shown promise for delivering iNOS inhibitors in preclinical TNBC models [212]. Second, validating RNS-based predictive biomarkers in large, multicenter clinical studies is essential to enable patient stratification: confirming that biomarkers such as iNOS expression levels, ONOO^−^ accumulation, or L-arginine metabolism flux can identify patients most likely to benefit from RNS-targeted therapies (e.g., patients with high iNOS expression responding to iNOS inhibitors) will ensure that these therapies are used in the right patient populations [213]. Finally, establishing standardized protocols for measuring RNS levels in clinical samples (e.g., tumor biopsies, blood, or cerebrospinal fluid) using high-sensitivity detection methods will address the current lack of consistency across studies, allowing for more reliable comparisons of RNS status between different patient cohorts and facilitating the integration of RNS biomarkers into clinical decision-making [214].

## 8. Conclusions

Reactive nitrogen species (RNS) form a multidimensional regulatory network in the tumor microenvironment (TME) through three core mechanisms: autonomous generation by tumor cells (via hypoxia/HIF-1α and inflammation/NF-κB-driven iNOS upregulation) and immune cells (e.g., TAMs and TANs via TLR-mediated iNOS activation), intracellular modulation of key tumor cell functions (proliferation, apoptosis, invasion, and metabolic reprogramming), and intercellular signaling interactions with immune, stromal, and vascular components. This network is not only central to tumor initiation and progression but also provides a wealth of translational targets, from RNS-specific diagnostic probes (e.g., BODIPY for NO, near-infrared probes for ONOO^−^) to therapeutic agents (e.g., the iNOS inhibitors 1400W and AG, the GST-activated NO prodrug JS-K). Preclinical studies have demonstrated the efficacy of these approaches – for example, 1400W reduces tumor growth and metastasis in TNBC models, while JS-K induces apoptosis in CRPC cells – supporting the potential of RNS modulation for precision oncology. By leveraging RNS-associated biomarkers to guide personalized interventions (e.g., iNOS inhibitors for high-RNS tumors, NO donors for low-RNS tumors), RNS-targeted strategies can address the heterogeneity of cancer and improve patient outcomes.

## Abbreviations

The following abbreviations are used in this manuscript:

**Table:** 

α-KG	α-Ketoglutarate
ADCC	Antibody-Dependent Cellular Cytotoxicity
AIF	Apoptosis-Inducing Factor
AKT	Protein Kinase B
AMPK	AMP-Activated Protein Kinase
ARG1	Arginase-1
ARNT	Aryl Hydrocarbon Receptor Nuclear Translocator
BET	Bromodomain and Extra-Terminal Motif
CAFs	Cancer-Associated Fibroblasts
CaM	Calmodulin
CaMKK2	Calcium/Calmodulin-Dependent Protein Kinase Kinase 2
CDK	Cyclin-Dependent Kinase
CTLs	Cytotoxic T Lymphocytes
Cyt c	Cytochrome c
CTCs	Circulating Tumor Cells
DR-5	Death Receptor 5
ECM	Extracellular Matrix
EPCs	Endothelial Progenitor Cells
EPR	Electron Paramagnetic Resonance
ESIPT	Excited-State Intramolecular Proton Transfer
eNOS	Endothelial Nitric Oxide Synthase
Fas	Fas Cell Surface Death Receptor
Fas-L	Fas Ligand
GEFs	Guanine Nucleotide Exchange Factors
GDH	Glutamate Dehydrogenase
GLUT1	Glucose Transporter 1
GLS1	Glutaminase 1
HIF-α	Hypoxia-Inducible Factor-1 Alpha
HK2	Hexokinase 2
HRE	Hypoxia-Responsive Elements
IF1	ATP Synthase Inhibitory Factor 1
IFN-γ	Interferon-Gamma
IL-1β	Interleukin-1 Beta
IL-6	Interleukin-6
IL-12	Interleukin-12
iNOS	Inducible Nitric Oxide Synthase
IRF5	Interferon Regulatory Factor 5
ICT	Intramolecular Charge Transfer
ITAM	Immunoreceptor Tyrosine-Based Activation Motif
JAK-STAT	Janus Kinase-Signal Transducer and Activator of Transcription
LPS	Lipopolysaccharide
MAPK	Mitogen-Activated Protein Kinase
MHC-II	Major Histocompatibility Complex Class II
MMP-2	Matrix Metalloproteinase-2
MMP-9	Matrix Metalloproteinase-9
MMP-13	Matrix Metalloproteinase-13
MDSCs	Myeloid-Derived Suppressor Cells
MOMP	Mitochondrial Outer Membrane Permeability
MPTP	Mitochondrial Permeability Transition Pore
MT1-MMP	Membrane-Type 1 Matrix Metalloproteinase
mTOR	Mammalian Target of Rapamycin
mtROS	Mitochondrial Reactive Oxygen Species
MPO	Myeloperoxidase
NF-κB	Nuclear Factor Kappa-Light-Chain-Enhancer of Activated B Cells
N₂O	Nitrous Oxide
NK	Natural Killer
NOS	Nitric Oxide Synthase
NO	Nitric Oxide
NO₂	Nitrogen Dioxide
NCRs	Natural Cytotoxicity Receptors
nNOS	Neuronal Nitric Oxide Synthase
ONOO^−^	Peroxynitrite
OVA	Ovalbumin
OXPHOS	Oxidative Phosphorylation
PARP	Poly(ADP-Ribose) Polymerase
PDE	Phosphodiesterases
PDK1	Phosphoinositide-Dependent Kinase 1
PKC	Protein Kinase C
PKG	cGMP-Dependent Protein Kinase G
PHD	Prolyl Hydroxylase Domain
pDCs	Plasmacytoid Dendritic Cells
PI3K	Phosphatidylinositol 3-Kinase
RNS	Reactive Nitrogen Species
ROCK	Rho-Associated Kinase
ROS	Reactive Oxygen Species
sGC	Soluble Guanylate Cyclase
SNAP	S-Nitroso-N-Acetylpenicillamine
STAT1	Signal Transducer and Activator of Transcription 1
TCA	Tricarboxylic Acid
TNF-α	Tumor Necrosis Factor-Alpha
TNFR1	Tumor Necrosis Factor Receptor 1
TNFR2	Tumor Necrosis Factor Receptor 2
TME	Tumor Metabolic Microenvironment
TLR	Toll-Like Receptor
TIMPs	Tissue Inhibitors of Metalloproteinases
TRAIL	Tumor Necrosis Factor-Related Apoptosis-Inducing Ligand
Tregs	Regulatory T Cells
TAMs	Tumor-Associated Macrophages
TANs	Tumor-Associated Neutrophils
VEGF	Vascular Endothelial Growth Factor
VEGFR2	VEGF Receptor 2
VE-cadherin	Vascular Endothelial Cadherin
VHL	Von Hippel–Lindau
